# Individual Chunking Ability Predicts Efficient or Shallow L2 Processing: Eye-Tracking Evidence From Multiword Units in Relative Clauses

**DOI:** 10.3389/fpsyg.2020.607621

**Published:** 2021-01-15

**Authors:** Manuel F. Pulido

**Affiliations:** Department of Spanish, Italian and Portuguese, Center for Language Science, Penn State University, University Park, PA, United States

**Keywords:** chunking ability, individual differences, eye-tracking, L2 proficiency, multiword units, second language, processing, generalization

## Abstract

Behavioral studies on language processing rely on the eye-mind assumption, which states that the time spent looking at text is an index of the time spent processing it. In most cases, relatively shorter reading times are interpreted as evidence of greater processing efficiency. However, previous evidence from L2 research indicates that non-native participants who present fast reading times are not always more efficient readers, but rather shallow parsers. Because earlier studies did not identify a reliable predictor of variability in L2 processing, such uncertainty around the interpretation of reading times introduces a potential confound that undermines the credibility and the conclusions of online measures of processing. The present study proposes that a recently developed modulator of online processing efficiency, namely, chunking ability, may account for the observed variability in L2 online reading performance. L1 English – L2 Spanish learners’ eye movements were analyzed during natural reading. Chunking ability was predictive of overall reading speed. Target relative clauses contained L2 Verb-Noun multiword units, which were manipulated with regards to their L1-L2 congruency. The results indicated that processing of the L1-L2 incongruent units was modulated by an interaction of L2 chunking ability and level of knowledge of multiword units. Critically, the data revealed an inverse U-shaped pattern, with faster reading times in both learners with the highest and the lowest chunking ability scores, suggesting fast integration in the former, and lack of integration in the latter. Additionally, the presence of significant differences between conditions was correlated with individual chunking ability. The findings point at chunking ability as a significant modulator of general L2 processing efficiency, and of cross-language differences in particular, and add clarity to the interpretation of variability in the online reading performance of non-native speakers.

## Introduction

For several decades, psycholinguistic studies on first (L1) and second language (L2) processing have employed measures of reading times as an indicator of ease of processing. This connection rests on the “eye-mind assumption” (Just and Carpenter, 1980), i.e., the notion that the amount of time spent reading a word is representative of the time spent processing it. Although the eye-mind association is now known to be more complex than previously held (Miller, 2015), the basic rationale remains valid and is the foundation of a vast body of literature focused on processing (for methodological reviews of L2 processing studies see, e.g., Frenck-Mestre, 2005; Dussias, 2010; Jiang, 2015; Keating and Jegerski, 2015; Marsden et al., 2018). Longer reading times in psycholinguistic studies are typically associated with processing costs induced by relatively more difficult conditions; while faster reading times are associated with relative ease of processing in less demanding conditions.

However, in spite of these robust and well-documented effects, previous evidence has indicated that faster reading does not always index processing efficiency (Broughton et al., 2010; Kaan et al., 2015; Miller, 2015). For example, readers often take fast-reading strategies that favor “good-enough” interpretations (Karimi and Ferreira, 2016; Metzner et al., 2017), even if this comes at the expense of misinterpreting the input (e.g., sentences with non-canonical syntactic structures) (Ferreira et al., 2001; Ferreira, 2003; Ferreira and Patson, 2007). While more engaged individuals may also read faster (Broughton et al., 2010), fast reading times may be simply due to lack of engagement or to a good-enough approach to process information. This point has become perhaps even more evident with recent methodological advances. In a recent experiment, Metzner et al. (2017) examined fixation-related brain potentials time-locked to eye movements during natural reading. The co-registration data in Metzner et al. (2017) provide perhaps the first direct evidence of shallow processing at the neurophysiological level that is coupled with faster-than-expected reading behavior.

As in studies with native speakers, there is good evidence that L2 readers may sometimes take similar speed-favoring strategies even when this compromises comprehension and the ability to process the input effectively, resulting in shallow processing (Felser et al., 2003; Roberts and Felser, 2011; Kaan et al., 2015). The evidence of shallow reading in L2 speakers led Clahsen and Felser (2006) to propose the shallow structure hypothesis (SSH). The SSH suggests that L2 users may strategically focus on lexical, pragmatic and other surface-level cues to achieve efficient processing that may be on par with L1 performance (Clahsen and Felser, 2006). Others (e.g., Hopp, 2010, 2013) have specifically proposed that using the L2 poses greater demands on the cognitive system and limits the resources available when using the less-dominant language. For example, the lack of automatization in lexical retrieval during early stages of processing may cascade into difficulties in forming syntactic representations in real time (Hopp, 2015). This latter perspective may also account for shallowness of processing in some of the participants of L2 processing studies, who fail to show the effects of experimental manipulations in comparisons between conditions. While it is not the goal of this paper to adjudicate between the different accounts, it does make a strong prediction about the availability of cognitive resources as a factor critically modulating individual L2 processing performance.

When reading in a non-native language, processing costs may be exacerbated as a result of lower L2 proficiency (Kotz and Elston-Güttler, 2004; Jeon and Yamashita, 2014), as well as lack of syntactic-semantic congruency between the L1 and L2 (Tokowicz and MacWhinney, 2005; Foucart and Frenck-Mestre, 2011; Wolter and Gyllstad, 2011), and slower lexical access (Hopp, 2013, 2015), among other issues. While such difficulties are usually associated with longer reading times, this is not always the case. Two important findings from previous research are that (a) strikingly, some L2 users may read even faster than control native speakers, exhibiting implausibly short reading times that indicate shallow reading (e.g., Experiments 2 and 4 in Felser et al., 2003; Kaan et al., 2015); and that (b) when matched in proficiency, faster non-native readers may present smaller differences between conditions and reduced grammaticality effects, suggesting shallower parsing (Kaan et al., 2015).

If faster reading times can index processing that is deep, engaged and efficient, but also processing that is shallow, disengaged and inefficient, it is clear that this creates an important confound which critically affects the interpretation of reading times in psycholinguistic studies^1^. It is still unknown what specific factors predict when some readers will show faster or slower reading times in the L2. Language proficiency has long been known to be a strong predictor of reading comprehension in an L2 (Alderson, 1984, 1993; Bowey, 1986; Koda, 2005; Kieffer and Lesaux, 2008, 2012a,b; Shiotsu, 2010; for a recent meta-analysis see Jeon and Yamashita, 2014). While lower linguistic skills in non-native speakers are typically associated with slower reading times, especially when compared with L1 speakers (Coughlin and Tremblay, 2012), online measures have yielded mixed evidence on whether reading speed correlates with proficiency (Roberts and Felser, 2011; Kaan et al., 2015). In other words, there is a lack of understanding of the modulators driving the strategic deployment of resources during reading, which may be able to account for and predict L2 reader performance. Clarifying the underlying modulators of L2 processing should be a priority, if potential confounds are to be avoided in the conclusions drawn from reading experiments. However, in spite of these findings, most studies have continued to straightforwardly consider faster reading times as a hallmark of efficiency in processing.

A specific cause of concern is the possibility that, in at least some cases, such low-efficiency readers may make up a non-negligible proportion of the sample. If correct, this implies that shallow reading is, at the very least, a likely contributor of noise in the data and a cause of a potential confound in the conclusions reached. That is, linguistic and cognitive measures are believed to modulate reading times but, based on previous evidence, I propose that this relationship may be non-linear: Individuals with higher L2 and/or cognitive skills should tend to present smaller differences between experimental conditions as well as faster reading times. However, a similar pattern is expected from individuals with low cognitive resources and low proficiency, if they only engage with the input at a shallow level. This may produce an inverse U-shaped curve, with relatively faster reading times at each end of the processing-efficiency continuum, even if for entirely different reasons.

To address this hypothesis, the present research examines whether individual differences in linguistic and cognitive abilities that support online processing may account for the variability in reading speed among L2 readers. To do so, it considers the role of L2 lexical knowledge in conjunction with chunk sensitivity, i.e., a recently developed cognitive measure found to be a significant predictor of processing efficiency, and a modulator of online reading (McCauley and Christiansen, 2015; McCauley et al., 2017; López-Beltrán et al., 2020). While a number of well-known measures of cognitive skill have been investigated, their predictive power in what concerns online processing appears to be limited. For example, greater engagement of executive control has been found to be associated with efficiency in recovery from initial misinterpretations, both in the L1 (Novick et al., 2013; Hsu and Novick, 2016) and in the L2 (Navarro-Torres et al., 2019). However, the role of executive control in such studies is most theoretically relevant in cases in which conflicting representations require controlled selection, specifically, rather than in processing across the board. Other previous studies have often focused on the role of working memory (WM) in reading. There is ample evidence that WM capacity predicts outcomes in offline reading comprehension both in the first (Baddeley, 1979; Daneman and Carpenter, 1980; Siegel, 1994) and in the second language (Harrington and Sawyer, 1992; Payne et al., 2009; Sagarra and Herschensohn, 2010; Erçetin and Alptekin, 2013; Hopp, 2014). However, WM appears to play a small role in measures of online L2 processing (Juffs, 2004; Havik et al., 2009; Roberts, 2012; Hopp, 2014; López-Beltrán et al., 2020; although see Navarro-Torres et al., 2019).

Recent evidence has suggested that better chunking ability is associated with more efficient online processing in native (McCauley and Christiansen, 2015; McCauley et al., 2017) and non-native speakers (López-Beltrán et al., 2020). Briefly, previous work has proposed that in order to deal with the immediacy of language, speakers must be sensitive to the structural probabilities in the input, if they are to successfully process the linguistic signal in real time (“the now-or-never bottleneck”; Christiansen and Chater, 2016). Based on well-known cognitive constraints that limit the amount of input information that can be maintained in memory (Miller and Taylor, 1948; Elliott, 1962; Pashler, 1988; Remez et al., 2010), Christiansen and Chater (2016) proposed that chunking may play a critical role in facilitating real-time processing, by allowing humans to recode the incoming signal into chunks at multiple levels of abstraction (from phonemes, to words, phrases and sentences). To illustrate, while recalling a sequence such as *h c r l t i a p a c e a p* poses a considerable challenge, the task becomes easier when the same string is re-arranged into a sequence of recognizable words, as in *c a t a p p l e c h a i r.* Given previous claims that non-native speakers may not always process the input efficiently in real time, the role of chunk sensitivity may be a particularly relevant predictor of online L2 comprehension. The present study will assess the role of chunking ability as a potential predictor of online processing and reading speed. To do this, measures of chunk sensitivity will be collected from L1 English—L2 Spanish learners using the tasks available in each of those two languages. If, as suggested by some accounts (Hopp, 2006, 2010; Kaan et al., 2015), differences in the resources available to L1 and L2 speakers are responsible for reading patterns, chunking ability may indicate, as an index of L2 processing efficiency, whether relevant linguistic cues are in fact processed online. An important question, however, is whether L2 processing is best predicted by chunk sensitivity measured in the L1 or the L2. So far, this question has only been explored in one self-paced reading study (López-Beltrán et al., 2020). Using eye-tracking, the present study will investigate the potential effect of individual chunking ability as online L2 sentence processing unfolds.

## The Present Study

To investigate individual variability in L2 reading, this study will capitalize on processing costs induced by multiword units that are incongruent across the native and non-native language, as a tool to investigate the processing of information that is particular to the L2. It therefore builds on a considerable number of studies that have identified, within the last decade, L1-L2 incongruent multiword units as a locus of processing costs (e.g., Yamashita and Jiang, 2010; Wolter and Gyllstad, 2011, 2013; Carrol et al., 2016; Wolter and Yamashita, 2018; Pulido, 2020).

Multiword units that are congruent (i.e., have word-by-word equivalents) are known to experience a processing advantage (even when encountered for the first time, e.g., Carrol and Conklin, 2017). On the other hand, L1-L2 incongruent multiword units, which differ at least in part from their L1 counterparts, are notoriously difficult to acquire (e.g., Nesselhauf, 2003; Laufer and Girsai, 2008; Pulido and Dussias, 2020) (e.g., in Spanish *pedir una hamburguesa* is equivalent to “order a hamburger,” but it literally translates as “request a hamburger”). A number of studies have consistently found cross-linguistic costs in processing of L1-L2 incongruent multiword units such that, even when these are well known, they produce costs in L2 processing (Yamashita and Jiang, 2010; Wolter and Gyllstad, 2011, 2013; Wolter and Yamashita, 2018). Therefore, L1-L2 incongruent collocations provide an ideal testbed to investigate variability in L2 reading based on L2 experience and individual differences in processing efficiency. In the present study, participants will be presented with multiword units composed of a verb and a noun. In addition, while the previous studies investigating cross-language effects on collocational processing employed reaction time tasks (such as lexical decision or phrase acceptability judgments), the experiment reported here examines online processing during natural reading.

An eye-tracking reading task is employed to examine learners’ processing of L2 multiword units, half of which are L1-L2 incongruent, e.g., *pedir una hamburguesa* (“order a hamburger”). The sentences included a relative clause in which the verb-noun unit was reversed, so that the incongruent element (i.e., the verb) would be focalized after the noun (e.g., *Él dice que las hamburguesas que pedirán son las mejores de la ciudad*, “He says that the hamburgers they will order are the best in town”). While previous data indicates that whether a multiword unit is presented in the canonical or a non-canonical order affects its recognition speed (e.g., in binomials; Siyanova-Chanturia et al., 2011b), in the present design this manipulation affects all target items equally. Importantly, eye-tracking data has indicated that the properties of multiword units are retained even when reversed (e.g., an idiom advantage is shown by “the bucket was kicked”, based on the canonical “kick the bucket”; Kyriacou et al., 2020). In this line, the L1-L2 congruency effect of collocations is expected to remain unaffected. Thus, data from eye movements collected during natural reading will allow to investigate the effect of individual-based differences in processing efficiency and speed during natural reading, in conditions of high ecological validity.

Based on previous findings, in the present study I hypothesize that online L2 reading is modulated by individual linguistic and cognitive skills. Specifically, in the approach taken here, rather than dividing the sample into overall slow/fast readers as in some previous studies (e.g., Hoover and Dwivedi, 1998; Roberts and Felser, 2011; Kaan et al., 2015), L2 multiword knowledge and individual chunking ability are examined as potential factors driving differences in reading times.

### Research Questions

RQ1. In what concerns processing efficiency, how do chunking ability measures (in the L1 and/or the L2) modulate reading times?

RQ2. In connection with L2 proficiency, how does command of L2-specific multiword knowledge modulate reading measures?

RQ3. Do chunking ability and L2 proficiency (indexed by L2 multiword unit knowledge) interact to modulate reading times and, if so, in what manner?

RQ4. In what concerns cross-linguistic differences, how do these measures differ or converge during processing of L2 multiword units that are congruent with the L1 equivalents, or L1-L2 incongruent?

RQ5. Based on eye-tracking measures, at what stage of processing will individual differences in processing become apparent? Specifically, how do L2 multiword knowledge and chunking ability affect eye movements in early measures (i.e., gaze duration) and late measures (i.e., total reading times)?

### Predictions

If faster reaction times in previous studies are associated with more efficient processing in some readers, but also with shallow processing in individuals with lower chunking ability, one would expect an inverse U-shaped pattern in the effect of chunking ability on reading speed (RQ1). If this prediction is correct, the research questions will clarify the contribution of each factor to modulate reading times of L2 multiword units, when these are congruent and incongruent with their L1 counterparts. Regarding the two versions of the Chunk Sensitivity task (in the L1 and the L2), previous results from self-paced reading data suggest that L2 processing might be predicted by chunking ability measured in the L1.

Regarding the role of L2 proficiency, the evidence from previous studies is mixed (RQ2). On the one hand, higher proficiency should be correlated with faster reading speed. At lower proficiency levels, slower reading times would be typically expected. However, knowledge of L2-specific features may interact with chunking ability in complex ways (RQ3). In particular, individuals at the low end of both L2 multiword units and chunking ability may be particularly prone to engage with the input at a shallow level.

The prediction for RQ4 is that, in reading V-N phrases, items that L1-L2 incongruent should result in greater costs (e.g., Yamashita and Jiang, 2010; Wolter and Gyllstad, 2011, 2013). More specifically, cross-language effects should emerge at elements that differ across the L1 and L2 (e.g., the verb in phrases such as “las hamburguesas que pedirán,” as in the example above). But no cross-language effects should emerge at the nouns, which are congruent across the L1 and the L2.

Finally, at least one previous study has found that both early and late measures were sensitive to the degree of conventionalization of V-N multiword units (Vilkaitė, 2016). Given important differences between the present study and the stimuli of previous studies, no specific predictions are made in what concerns early versus late measures of processing, or their possible interaction with the other variables investigated. Thus, the measures of gaze duration and total duration will be examined with no strong *a priori* expectations.

## Materials and Methods

### Participants

A group of 45 participants was recruited at The Pennsylvania State University. Participants were native speakers of English who were enrolled in third and fourth semester university Spanish courses (roughly equivalent to level B1 of the Common European Framework of Reference for Languages, Council of Europe, 2011). This sample size is comparable to that of previous studies that have consistently detected cross-language effects during processing of L2 multiword units (Yamashita and Jiang, 2010; Wolter and Gyllstad, 2011, 2013), and is in line with recent studies that examined individual-based differences in chunking ability, both in the L1 (McCauley et al., 2017) and the L2 (López-Beltrán et al., 2020)^2^.

Participants completed a linguistic background questionnaire as well as proficiency measures to confirm they met the required proficiency level. One subject who was identified as an early bilingual was excluded. Five additional participants were excluded due to accuracy lower than 70% during the reading task (*N* = 1), due to experimental error during data acquisition (*N* = 1), or because they failed to complete all the sessions (*N* = 3). The results and data analysis reported below are based on the remaining thirty-nine participants (77% female). The study was approved by the Institutional Review Board; all participants gave informed consent and were paid 10 US dollars per hour of participation.

### Materials and Procedure

**FIGURE 1:**
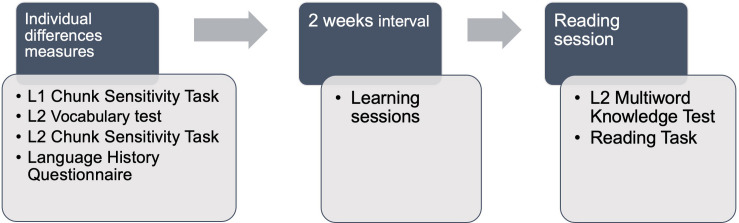
Illustration of task sequencing in experimental protocol.

In a first session, participants completed individual differences measures in this order: English Chunk Sensitivity, Spanish Vocabulary test, Spanish Chunk Sensitivity task and Language History Questionnaire; L2 tasks were blocked this way to avoid switching repeatedly between the L1 and L2, while separating the two versions of the Chunk Sensitivity task. Participants then completed three sessions as part of a multiword unit learning study that will be reported elsewhere (Pulido, 2020). Two weeks after the first session, participants returned to the lab to complete the Multiword Units test and the Reading task. This way, multiword unit knowledge was tested immediately before the main Reading task. The sequencing of tasks is illustrated in Figure 1.

For ease of presentation, this section first describes the materials created for the Reading task, followed by the materials of individual differences tasks employed to measure chunking ability and language proficiency.

#### Reading Task Materials and Procedure

##### List of V-N multiword units

First, a list of thirty-eight target V-N phrases was created, half of which were congruent (i.e., had literal equivalents) across English and Spanish, while the other half were cross-linguistically incongruent, i.e., specific to the L2. While phrases congruent with the native language are interpretable based on cross-language similarity (Siyanova-Chanturia et al., 2011a; Pulido and Dussias, 2020), incongruent items require knowledge specific to the L2. Given this, incongruent items were selected based on their similarity to a list of collocations that had been recently learned by the participants. For example, based on the collocation *pedir pizza* (“request pizza,” equivalent to “order pizza”) previously learned by the participants, the related V-N phrase *pedir una hamburguesa* (“order a hamburger”) was created. This made all target phrases employed here interpretable based on prior knowledge.

Furthermore, to ensure the interpretability of incongruent multiword units, the semantic relatedness between the nouns in the previously learned and the current incongruent target phrases was measured through PMI (pointwise mutual information) (Budiu et al., 2007; Bullinaria and Levy, 2007; Recchia and Jones, 2009). PMI has been shown to be a valid index of semantic relatedness, and to even be more highly correlated to human judgments than more computationally intensive measures such as Latent Semantic Analysis, when based on large corpora (>400 million words, Recchia and Jones, 2009)^3^. Data from a large web-based corpus of over two billion words (Corpus del Español, Davies, 2016) was used to calculate the scores of similarity and confirm the semantic similarity between the nouns in previously learned and target phrases. To compare related target pairs to an unrelated baseline, the similarity scores of target pairs were compared against a list of unrelated pairs, consisting of the same words re-matched (e.g., *tren – pizza*, “train” – “pizza”). This confirmed that target items were semantically similar to known items (mean similarity score for fillers: −2.06; SD: 1.23; mean for related items: 2.63, SD: 2.33; *p* < 0.0001). The full set of phrases (including the related previously learned incongruent items) are available in the Supplementary Material.

Importantly, in all incongruent phrases, the verb was the element that differed across the L1 and the L2, while the noun was literally congruent. Lists of congruent and incongruent phrases were matched on properties including the length and log frequency of verb and the noun; per-million observations of verb-noun multiword units; and verb-noun association strength (t-scores). Given that the focus was on processing of the verbs, and in order to minimize comprehension problems in reading novel phrases, nouns in all V-N phrases were cognates.

##### Reading materials

Based on each multiword unit from the list described above, a preamble and a carrier sentence were created, which were followed by a comprehension question. The preamble was included to provide context, and the ensuing carrier sentence contained the target multiword unit. The length of preambles and target carrier sentences was not significantly different for trials containing congruent and incongruent multiword units (all *p* > 0.50). Half of the comprehension questions items (50% true, 50% false) were related to the content of the preamble and the other half to the carrier sentence. A full trial is illustrated in Table 1. The full list is available in the Supplementary Material.

**TABLE 1:** Sample materials for one experimental trial.

**Preamble**
Juan planea invitar a algunos amigos a su restaurante favorito
(“Juan plans to invite some friends to his favorite restaurant”)
**Target sentence**
Él dice que las hamburguesas que *pedirán* son las mejores de la ciudad
(“He says that the hamburgers that they *will order* are the best in town”)
**Comprehension question**
¿Él recomienda la fruta?
(“Does he recommend the fruit?”) (Response: False)

*In these examples, target multiword units appear underscored, with verbs shown in italics (no text enhancement was used during the experiment).*

As indicated, all units were composed of a verb and a noun. However, carrier sentences included a relative clause in which the V-N phrase was reversed, so that the incongruent element (i.e., the verb) would follow the noun. To illustrate, based on the V-N phrase *pedir hamburguesas* “order hamburgers,” a carrier sentence was created: *Él dice que las hamburguesas que pedirán son las mejores de la ciudad* (“He says that the hamburgers that they will order are the best in town”). Given that Spanish and English have virtually identical syntax in subject relative clauses, the syntactic frame itself is cross-linguistically congruent (i.e., Spanish N-“*que*”-V, English “N-[*that*]-V”)^4^. Further, although as in English, subject relative clauses are “non-canonical” relative to the V-N order in Spanish, they are very highly productive. For example, while “pedir” + “hamburguesa” in the V-N order occurs 131 times in the Corpus del español (4 word span), the inverted N-V order also occurs 17 times, in a ratio of about 1 to 8.

This manipulation served several goals. First and foremost, given the focus on verbs (which are L1-L2 incongruent in half of the items), the relative sentence structure allows to present a verb that is maximally predictable based on the preceding context. That is, unlike in a V-N order of presentation, seeing the noun (direct object) first means that the verb is fully interpretable as soon as it is encountered, relative to cases in which the verb’s object is presented later in the sentence. Secondly, because participants had previously learned verb-noun collocations in the canonical word order as part of a larger project, this context allows to examine the role of chunking ability independently from prior practice with a given syntactic frame. Third, it extends previous research which examined the effect cross-language congruency when reading V-N phrases to sentences containing relative clauses.

##### Reading task procedure

Participants’ eye movements were recorded using an Eyelink 1000 plus eye-tracker (SR Research, Inc). Eye movements were recorded from the right eye. Participants were seated at 90 cm from the monitor and comfortably rested their chins on a chin rest. At the start of the experiment, a nine-point calibration procedure was performed, followed by a calibration accuracy test. Calibration was checked at the beginning of each trial, and was repeated if any point had an error greater than 1°, or if the average error for all points was greater than 0.5°. Each sentence was presented left-aligned in the center of the screen. Text was displayed in Consolas size 15 font. To begin each trial, participants looked at a fixation point coinciding with the first character position of the sentence. Participants were instructed to read each sentence at their own pace, and to press a button to advance to the next screen. The preamble and target sentences appeared in separate screens in one single line; therefore, the target multiword phrase was never displayed at the beginning or end of the line. At the end of the trial, participants responded to the question by pressing a “yes” or a “no” button on a hand-held device. Four practice trials preceded the experimental items. The task lasted approximately 25 min.

#### Individual Differences Measures

Participants completed a measure of Chunk Sensitivity in both of their languages (L1-English and L2-Spanish) as well as additional measures to assess short-term memory and language proficiency in the L1 and L2. The measures are described below and their outcomes are reported in the section “Results.”

##### Chunk sensitivity tasks

Participants’ individual chunking ability was measured using two versions of the Chunk Sensitivity task, each in one of participants’ languages (L1 English and L2 Spanish). In this task, participants are instructed to recall strings of 12 individual words, each made up by 4 trigrams. The task includes 20 strings, evenly divided into target and control trials. Each target trial consists of four frequent trigrams extracted from native speaker corpus data, which lend themselves to be “chunked” as a unit (e.g., have to eat good to know don’t like them is really nice). Matched controls are made up of unrelated words with no statistical association (e.g., years got don’t to game have she mean to them far is), and thus provide a baseline for short-term memory span. A chunk sensitivity index is calculated as the difference in recall accuracy between targets and controls.

Responses were recorded and coded offline. Each correctly recalled word in a string is awarded 1 point, for a maximum of 3 points per trigram (12 points for the whole string). One point is deducted from the whole trial for imperfect ordering.

The English version employed here contained the materials in the original task reported in McCauley et al. (2017), which was created based on data from the American National Corpus (Reppen et al., 2005) and the Fisher corpus (Cieri et al., 2004). The Spanish version was developed by López-Beltrán et al. (2020), based on data from the *Corpus del español* (Davies, 2016); the complete description of the tasks is reported in López-Beltrán et al. (2020).

##### Phonological short-term memory

While the chunking ability tasks are designed to factor out the role of phonological short-term memory (PSTM) from recall of multiword chunks, previous research has shown that PSTM is on its own a significant predictor of learners’ ability to retrieve L2 collocations (Pulido and Dussias, 2020). Therefore, participants were administered a Non-word Repetition task (Baddeley et al., 1988) as an index of PSTM. The lists employed here were based on the materials reported in Martin and Ellis (2012). Four lists of three, four, five or six non-words were presented in ascending order. Participants’ responses were recorded and were scored offline following a scheme adapted from Gathercole et al. (2001). One full point was awarded for each correctly recalled non-word (up to a maximum of 72). For partially correct non-words, 0.25 was deducted for each error in the position of a phoneme; and 0.5 was deducted for each missing phoneme, or for phonemes that were not part of the trial.

##### Language experience and proficiency measures

To assess the linguistic profile and background in the L1 and L2, participants completed the LEAP-Q (Marian et al., 2007), which contained items related to weekly usage and exposure to the L2.

##### Vocabulary test

Prior word knowledge was measured through a multiple-choice vocabulary test. While knowledge of L2 multiword units was important to the goals of the study and measured separately, the rationale for this test was to assess participants’ knowledge of the basic meaning of individual verbs and nouns, outside the scope of L2 multiword units. Items consisted of the basic meaning of 84 items, including the nineteen verbs used in the L1-L2 incongruent multiword units; for these units, the basic word meaning assessed in the test (e.g., “pedir” = “request”) differs from its specialized meaning in an L2-specific collocation (as described, “pedir hamburguesas” literally translates as “request hamburgers” but is equivalent to “order hamburgers”). Therefore, the test allowed to gauge individual word knowledge, as opposed to knowledge of multiword units; this was considered important because a learner might know the basic meaning of the word, even if the specialized meaning is less familiar^5^. The test materials are available in the Supplementary Material.

##### L2 multiword knowledge test

A L2 multiword unit test measured participants’ knowledge of previously learned incongruent V-N collocations as part of a larger project (reported in Pulido, 2020), and was administered right before the reading experiment [see section “Reading Task Materials and Procedure” on how the current materials were related to the learned items]. The L2 multiword knowledge test, which followed previous learning of L1-L2 incongruent collocations as part of a larger study, provided a baseline individual measure of L2-specific multiword knowledge. Participants were presented with L1 verb-noun phrases and were asked to provide the previously learned L2 translation. To clarify, it tested multiword unit knowledge by using items which were not included in the present study. Knowledge of multiword units is the critical aspect of L2 proficiency under examination, and this measure is referred to as “L2 multiword knowledge.”

### Data Cleaning and Analysis

The reading measures reported are gaze duration and total reading times. The analysis reported here follows recent studies on reading of Verb-Noun collocations that examined processing using a combination of early (gaze duration) and late measures (total duration) (e.g., Vilkaitė, 2016; Vilkaite and Schmitt, 2019). Gaze duration is defined as the sum of all eye-fixations on the critical region of interest before leaving it the first time that it is read; total times are the sum of all fixations on the critical region, including regressions (re-reading). As described, each Verb-Noun phrase (e.g., *pedir hamburguesas* “order hamburgers”) was presented in a relative sentence, e.g., *las hamburguesas que pedirán* “the hamburgers that they will order.” Gaze and total durations were extracted for reading times of the verb (in the previous example, *pedirán* “they will order”), where a stronger effect was expected; as well as for the preceding noun phrase (*las hamburguesas* “the hamburgers”), which served as a baseline. While no effects were expected in the noun region for gaze duration (i.e., for fixations made prior to reading the verb), potential effects might be present for the measure of total duration, which includes regressions to the noun (e.g., after reading the verb).

Only trials with correct comprehension responses were included in the analysis. One item in the incongruent condition was excluded due to experimental error^6^. All participants included in the analysis met the threshold of 70% accuracy in comprehension (mean: 87.82% correct responses); one participant with low comprehension accuracy that was close to chance (57% correct) was removed. Inspection of the data revealed that, in transitioning from the preamble sentence to the target sentence, fixations were made on various parts of the sentence in some trials. To avoid repetition effects due to foveal and parafoveal processing of target regions in such cases, trials in which the first fixation was not on the first word of the sentence were removed (7.15%).

Total durations shorter than 100 ms or longer than 3000 ms were excluded (5.07%). The data were z-scored, and outliers were then removed for each condition based on their individual median absolute deviation (MAD) for each participant and condition. The MAD method is a more robust measure for outlier removal than standard deviations, given that the latter are susceptible to be distorted by observations that strongly deviate from the mean (Leys et al., 2013). Trials above or below 3 absolute deviations from the median were excluded, resulting in 3.27% data loss.

Separate analyses were conducted for each of the two dependent measures extracted (gaze duration and total duration times) for the verb and for the determiner-noun. That is, four separate models (2 measures × 2 interest areas) were developed following the same procedure. Data skewness in the dependent variable was corrected by log-normalizing the data^7^.

Mixed-effects modeling was conducted using the lme4-package (version 1.1-23; Bates et al., 2015) in R (version 4.0.2). Model fitting always started with a core model which included English and Spanish Chunk Sensitivity scores, L1-L2 Congruency and Collocation test scores (“L2 multiword knowledge”), to address the main research questions. Then the potential interaction and additional covariates were added to the model, which included: L1-L2 Congruency (congruent, incongruent), individual scores for L2 vocabulary and phonological short-term memory. Additionally, stimulus properties were considered, including log frequency and collocational strength (i.e., t-scores) of multiword units; as well as orthographic length and log frequency of the individual words in the region of interest. For models of the verb, the same variables were considered, but orthographic length and frequency of the preceding noun were also included. All continuous variables were centered. Variance Inflation Factors were calculated using the vif function of the car package (version 3.0-9; Fox et al., 2007), and indicated no substantial collinearity among the variables considered (all VIF < 2).

The random effects structure included by-subject and by-item random intercepts, as well as random by-subject slopes for trial number and L1-L2 Congruency, and by-item slopes for English and Spanish Chunk Sensitivity and previous Collocation test scores. Following convergence issues with the maximally specified structure (Barr et al., 2013), the random effects structure was simplified. All final models included by-subject and by-item random intercepts, as well as by-subject random slopes for trial number.

Starting from this full model, a backward step-by-step model selection process was adopted. Variables were removed one by one, starting with those with the lowest *t*-values. Predictors that did not significantly improve the model fit (likelihood ratio test *p* > 0.05) were removed. The code of each of the models is provided in the Supplementary Material. The following section reports on the results of the selected models. The results presented include 95% confidence intervals (CI) and parameter-specific *p*-values estimated using the normal approximation.

## Results

### Baseline Measures

The results of all the individual-based measures are reported below in Table 2. The scores from the baseline multiword units provided a broad proficiency range (range 42.9–96.4%), adequate for the goal of assessing the role of multiword-based proficiency along with chunking ability in processing. L1 and L2 Chunk Sensitivity were not significantly correlated (*r* = 0.15, *p* = 0.37). Similarly, neither measure was significantly correlated with the Multiword Units test scores (all *p*-values > 0.18). Cronbach’s alpha was calculated as a reliability index and found to be acceptable for all the tests (i.e., ranging from 0.70 to 0.90; Streiner, 2003; Tavakol and Dennick, 2011), including the vocabulary test (α = 0.75), the L2 multiword knowledge test (α = 0.80), the PSTM test (α = 0.86) and the Chunk Sensitivity tasks in the English (α = 0.86) and Spanish (α = 0.81) versions.

**TABLE 2:** Summary of cognitive and proficiency measures.

	Valid N	*M*	SD	Range
Age (in years)	39	18.76	0.85	18–21
Weekly exposure to L2 (%)	39	6.26	4.74	0–18
Baseline Vocabulary Test (/10)	39	8.64	0.61	7.1–9.8
L1 Chunk Sensitivity	39	37.72	12.43	10–58
L2 Chunk Sensitivity	39	13.72	7.72	−2–39
PSTM: Non-word repetition (/10)	37	6.12	0.99	3.7–8.0
L2 multiword knowledge (/10)	39	7.88	1.34	4.3–9.6

*All scores reported are based on a scale from 0 to 10, unless otherwise indicated. The Chunk Sensitivity measures are the difference scores between target and control trials (with up to 120 points in each condition).*

### Reading Times of the Noun Region

#### Gaze Duration (Noun Region)

As expected, the analysis of gaze duration for the noun revealed no significant effects of Chunk Sensitivity in the L1-English measure (β: −12.76, SE: 13.88, CI: −39.11, 13.59, *p* = 0.36), nor in the L2-Spanish version of the task (β: 15.69, SE: 13.81, CI: −10.45, 41.92, *p* = 0.26). There was also no significant effect of individual knowledge of L2 collocations (β: 3.77, SE: 13.55, CI: −22.22, 29.87, *p* = 0.78). Finally, as expected, cross-language Congruency did not significantly affect gaze duration of the noun baseline (β: 24.21, SE: 19.27, CI: −13.75, 62.25, *p* = 0.21). The results are shown in Table 3.

**TABLE 3:** Summary of selected models for the noun region.

	Gaze duration				Total duration			
Fixed Effects	β	*SE*	*t*	*p*	β	*SE*	*t*	*p*
(Intercept)	585.39	17.12	34.19	***	6.42	0.06	100.18	***
Eng. (L1) Chunk Sensitivity	–12.76	13.88	–0.92	0.36	0.03	0.04	0.89	0.38
Span. (L2) Chunk Sensitivity	15.69	13.81	1.14	0.26	–0.08	0.04	–2.07	*
L2 multiword knowledge	3.77	13.55	0.28	0.78	0.02	0.04	0.45	0.66
Congruency (incongruent)	24.21	19.27	1.26	0.21	0.12	0.08	1.55	0.12

**Random effects**	**Variance**	***SD***	**Correlations**		**Variance**	***SD***	**Correlations**	

Intercept | Participant	3542.7	59.52			0.05	0.22		
Trial Number | Participant	187.1	13.68	0.11		0.01	0.12	0.34	
Intercept | Item	596.5	24.42			0.05	0.21		

Marginal *R*^2^: 0.007, Conditional *R*^2^: 0.06					Marginal *R*^2^: 0.03, Conditional *R*^2^: 0.32			

*p < 0.05, **p < 0.01, ***p < 0.001.

#### Total Duration (Noun Region)

The results of total duration for the noun region indicated no significant effect of Chunk Sensitivity in the L1 (β: 0.03, SE: 0.04, CI: −0.04, 0.11, *p* = 0.38). There was also no significant effect of L2 multiword knowledge (β: 0.02, SE: 0.04, CI: −0.06, 0.09, *p* = 0.66), nor of L1-L2 Congruency (β: 0.12, SE: 0.08, CI: −0.04, 0.27, *p* = 0.12). However, there were significant effects of L2 Chunk Sensitivity, with higher Spanish chunking ability reducing total duration times (β:−0.08, SE: 0.04, CI: −0.15, −0.005, *p* < 0.05).

### Reading Times of the Verb Region

#### Gaze Duration (Verb Region)

**FIGURE 2:**
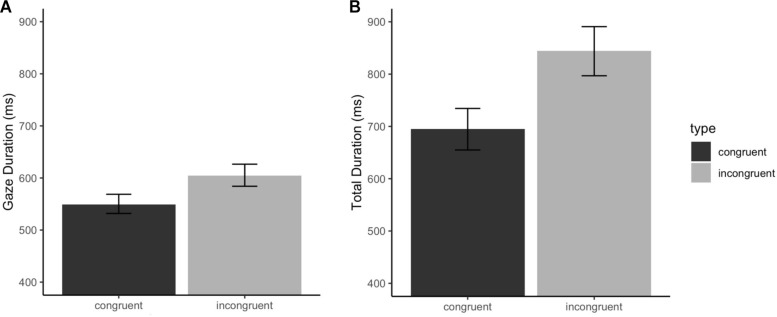
Effect of L1-L2 congruency of the verb on **(A)** gaze duration and **(B)** total duration reading times of the verb region. Error bars represent the 95% CI.

The analysis of gaze duration for the verb revealed a significant effect of L1 Chunk Sensitivity, such that greater chunking ability was associated with faster reading times (β: −28.05, SE: 12.77, CI: −52.56, −3.60, *p* < 0.05). However, L2 Chunk Sensitivity did not significantly influence the dependent measure (β: 18.06, SE: 12.21, CI: −5.16, 41.18, *p* = 0.14). There was a highly significant effect of L1-L2 Congruency, with slower reading times for incongruent trials relative to congruent trials (β: 58.38, SE: 17.97, CI: 23.02, 93.83, *p* < 0.01). Finally, gaze durations were influenced by the knowledge of L2 collocations and by vocabulary scores. Interestingly, these effects went in different directions, such that greater multiword knowledge was associated with longer gaze durations (β: 58.38, SE: 17.97, CI: 13.02, 61.17, *p* < 0.05), while higher knowledge of individual words reduced reading times (β: −27.61, SE: 13.04, CI: −52.17, −3.06, *p* < 0.05); these differences are further commented on in the discussion. The results are summarized in Table 4. The effect of verb cross-language congruency is illustrated in Figure 2.

**TABLE 4:** Summary of selected models for the verb region.

	Gaze duration				Total duration			
Fixed Effects	β	*SE*	*t*	*p*	β	*SE*	*t*	*p*
(Intercept)	545.92	15.56	35.07	***	6.33	0.06	99.3	***
Eng. (L1) Chunk Sensitivity	–28.05	12.77	–2.20	*	–0.04	0.05	–0.77	0.44
Span. (L2) Chunk Sensitivity	18.06	12.21	1.48	0.14	–0.07	0.05	–1.31	0.19
L2 multiword knowledge	36.99	12.34	3.00	**	0.07	0.05	1.46	0.14
Congruency (incongruent)	58.38	17.97	3.25	**	0.22	0.07	3.42	***
L2 Vocabulary	–27.61	13.04	–2.12	*				
L2 Chunk. × L2 Colloc.					0.00	0.06	0.06	0.95
L2 Chunk. × Congruency					0.07	0.03	2.06	*
L2 Chunk. × L2 Colloc. × Cong.					–0.09	0.04	–2.37	*

**Random Effects**	**Variance**	***SD***	**Correlations**		**Variance**	***SD***	**Correlations**	

Intercept | Participant	3219.65	56.74			0.07	0.27		
Trial Number | Participant	36.85	34.34	0.04		0.01	0.09	0.00	
Intercept | Participant	1179.28				0.03	0.18		

Marginal *R*^2^: 0.04, Conditional *R*^2^: 0.11		Marginal *R*^2^: 0.05, Conditional *R*^2^: 0.30						

*p < 0.05, **p < 0.01, ***p < 0.001.

#### Total Duration (Verb Region)

**FIGURE 3:**
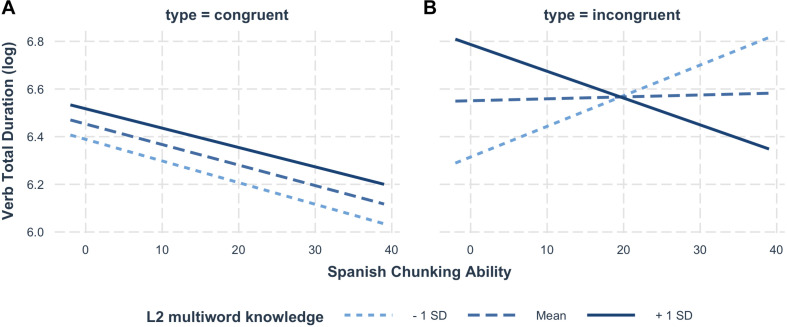
Illustration of the effect of three-way interaction between on **(A)** congruent and **(B)** incongruent multiword units. L2 multiword knowledge serves as the critical L2 proficiency measure.

Similarly to the results of gaze duration, the results of total durations for the verb revealed a highly significant effect of L1-L2 Congruency (β: 0.22, SE: 0.07, CI: 0.09, 0.36, *p* < 0.001). The analysis indicated no simple effect of Chunk Sensitivity in the L1 (β: −0.04, SE: 0.05, CI: −0.13, 0.06, *p* = 0.44) or in the L2 (β: −0.07, SE: 0.05, CI: −0.16, 0.03, *p* = 0.19). Importantly, a crucial significant three-way interaction emerged between L2 Chunk Sensitivity, L2 multiword knowledge and Congruency (β: −0.09, SE: 0.04, CI: −0.17, −0.02, *p* < 0.05). The two-way interaction between L2 Chunk Sensitivity and Congruency was also significant (β: 0.07, SE: 0.03, CI: 0.06, 0.14, *p* = 0.07). The *interactions* package in R (Long, 2019) was used to visualize and aid in interpreting these effects, which are illustrated in Figure 3; the interaction is examined in depth in the discussion. For congruent collocations, the data presented no interaction between the main effects of L2 chunking ability and multiword knowledge. Although higher chunking ability had a facilitatory effect, it is relevant to note that reading times were shorter for readers with less proficient L2-multiword knowledge. Table 4 summarizes the model output.

For incongruent collocations, critically, L2 chunking ability and multiword knowledge interacted, giving rise to an approximately inversed U-shape trend, such that the faster reading times were found in individuals with either low multiword knowledge and low chunk sensitivity, or with high multiword knowledge coupled with high chunk sensitivity. Interestingly, as can be observed in Figure 3, participants with lower multiword knowledge presented faster reading times, which became increasingly slower as chunking ability increased; for participants with average multiword knowledge, chunk sensitivity did not appear to strongly influence reading behavior; finally, individuals with higher multiword knowledge showed faster reading durations as chunking ability increased.

##### Follow-up analysis

**FIGURE 4:**
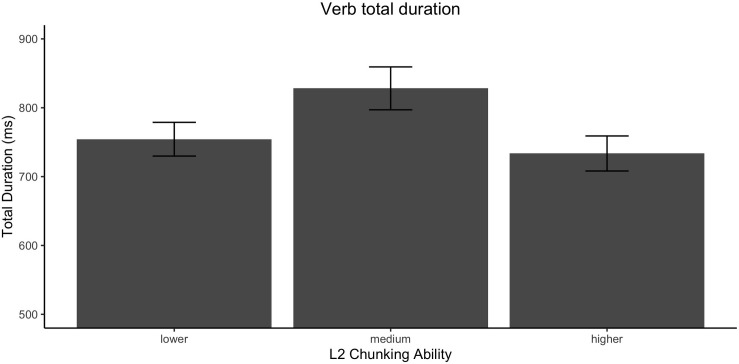
Effect of each tercile of L2 chunking ability on total duration times for the verb. Error bars represent the standard error.

Previous studies have performed a median split based on overall reading times, with the goal of further characterizing the individual variability within samples of L2 readers (e.g., Hoover and Dwivedi, 1998; Roberts and Felser, 2011; Kaan et al., 2015). However, as noted, in the present data on incongruent collocations faster reading times were found at both ends of the proficiency (i.e., L2 multiword knowledge) and chunk sensitivity measures. The results are indicative of the predicted inverse U-shaped trend at the sample level, where readers with higher and lower chunking ability present faster reading times, albeit for entirely different reasons. This is what is shown by the pattern illustrated in Figure 4; the division based on chunking ability confirms the prediction that high- and low-chunking ability readers have similar reading speed. As a result, a follow-up analysis that divided participants based on overall reading times might lump together high- and low-efficiency readers.

To further investigate the effect of L2 multiword knowledge and its interaction with Spanish Chunk Sensitivity, *post hoc t*-tests (FDR-corrected) were performed to gauge the effect of L1-L2 congruency within each subgroup of participants, based on chunking ability (high, medium, and low) and L2 multiword knowledge (i.e., high or low L2). The results in Table 5 provide further evidence that differences between conditions were driven by chunking ability, rather than knowledge of the L2 units. Crucially, high chunking ability readers presented a significant effect of congruency, regardless of L2 knowledge. On the contrary, readers with low chunking ability did not show congruency effects, also regardless of L2 knowledge.

**TABLE 5:** Results of the dedicated congruency-based pairwise comparisons.

L2 multiword knowledge	Chunking ability (tercile)	Congruent mean (ms) (SD)	Incongruent mean (ms) (SD)	*df*	*t*	*N*	95% CI	*p*
Low	Low	661 (474)	699 (504)	122.27	–0.61	5	−0.28, 0.15	0.55
Low	Medium	624 (472)	822 (550)	236.93	–3.54	8	−0.46, −0.13	**
Low	High	614 (349)	919 (635)	149.61	–3.61	6	−0.54, −0.16	*
High	Low	765 (452)	818 (474)	242.11	–1.06	8	−0.22, 0.07	0.44
High	Medium	1035 (739)	1050 (564)	112.07	–0.99	4	−0.37, 0.12	0.40
High	High	594 (316)	832 (592)	231.02	–3.37	8	−0.41, −0.11	**

*p < 0.05, **p < 0.01 (FDR-corrected values). Comparisons were performed on the log-transformed total reading times. The means of raw reading times are provided for ease of interpretation, with the standard deviation in parenthesis.

**FIGURE 5:**
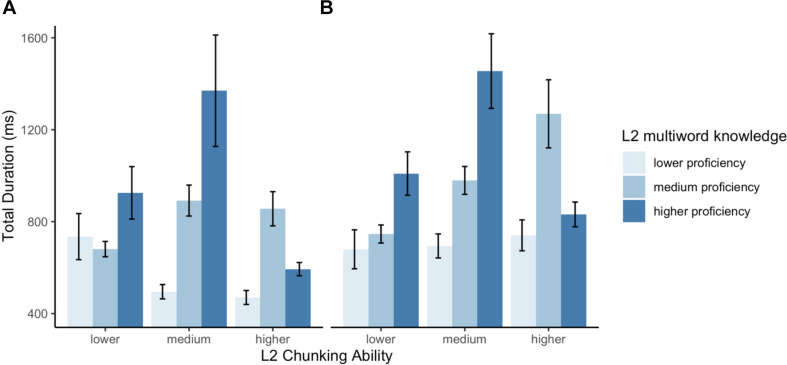
Effects of L2 chunking ability and multiword knowledge on verb total duration times for in **(A)** congruent and **(B)** incongruent multiword units. Levels of L2 chunking ability are represented in the horizontal axis, and levels of L2 multiword knowledge are shown in column colors. L2 multiword knowledge serves as the critical L2 proficiency measure. Error bars represent the standard error.

By way of visual illustration, Figure 5 further presents each variable split into the lower, medium and higher terciles (chunking ability is shown in the horizontal axis, with columns for each L2 tercile)^8^. Three main points should be noted. First, an inverse-U shape pattern can be observed for readers with high knowledge of L2-specific multiword units (darker columns), who could likely extract the most meaning from the target multiword units. This pattern is not present in the group with lower L2 multiword scores (lighter columns), who were likely to extract less meaning and process the input at shallow level. Secondly, the groups reveal that the readers with lower L2 scores (again, lighter columns) were homogeneously fast in processing the more challenging L1-L2 *incongruent* items, and were even faster than readers with both high-L2 chunking ability and high-proficiency. Third, critically, high chunking ability learners only account for a small fraction of the faster total reading times. In fact, the data reveal that a considerable portion of the shortest reading times can be attributed to the readers with lower chunking ability, and among those, to the subset with lower L2 multiword knowledge.

One might also wonder how these modulators affected offline reading comprehension. Although all reading time analyses were performed on accurate trials only, the effect of L2 multiword proficiency and chunking ability on accuracy in offline responses was examined for each tercile-based subgroup. Interestingly, and in contrast with online reading times, the results of pairwise *t*-tests indicated that accuracy in offline comprehension was not significantly modulated by chunking ability (all *p*s > 0.80), but it was modulated by L2 proficiency instead. Lower-proficiency readers (mean accuracy: 85.3%, SD: 7.28) had significantly worse offline comprehension (*p* < 0.01) than high-proficiency readers (mean: 93.8%, SD: 5.12), and medium-proficiency readers (mean: 90.6%, SD: 5.52; *p* < 0.05). This pattern further indicates a specialized role of chunking ability in predicting online processing efficiency, independent of other mechanisms that may affect the outcome of offline comprehension.

### Preamble Total Reading Times

Finally, one potential caveat affecting the generalizability of these results is that the focus of the analysis presented above was on N-that-V phrases and not on L2 reading more generally. To convincingly show the importance of chunking ability in reading, it would be necessary to demonstrate that this is a pervasive effect in sentence reading and not just on chunks (or modified chunks). The present data set allows to further investigate this question, by examining the overall reading times of the preamble sentences that preceded the critical sentences containing the multiword units^9^. Because the interest in this case is on the whole preamble sentence (and not on any specific manipulated region as in the critical sentences), the total reading times of the preamble were used as the dependent measure (mean: 6,974 ms; SD: 3,391 ms).

If Chunk Sensitivity does indeed modulate reading efficiency more generally, the reading times of the preamble should reveal similar effects as the ones observed for the analysis of the critical multiword units. Recall that in the total reading times analyses of both the noun and the verb regions, the L2 chunking ability measure emerged as a significant modulator of total reading times; therefore, a similar effect in the preamble would provide compelling evidence of a general effect in reading. Based on the same data cleaning and analytical procedures described above, a mixed-effect model analysis was performed on the log-transformed total reading times of the preamble. The same variables as in the main analysis were included, with the exception of variables specifically related to the N-that-V multiword units (as these were not part of the preamble); the selected model is available in the Supplementary Material.

Importantly, L2 chunking ability was again revealed to be a significant predictor (β: −0.11, SE: 0.05, CI: −0.20, −0.02, *p* < 0.05). The model output is presented in Table 6. The results of this analysis provide further support for a general role of chunking ability in L2 reading.

**TABLE 6:** Summary of model output for reading times of the preamble.

Fixed Effects	β	*SE*	*t*	*p*
(Intercept)	8.74	0.06	150.63	***
Eng. (L1) Chunk Sensitivity	0.04	0.05	0.90	0.38
Span. (L2) Chunk Sensitivity	–0.11	0.05	–2.40	*

**Random Effects**	**Variance**	***SD***	**Correlations**	

Intercept | Participant	0.10	0.32		
Trial Number | Participant	0.00	0.01	−0.49	
Intercept | Participant	0.05	0.22		

	Marginal *R*^2^: 0.05, Conditional *R*^2^: 0.69			

*p < 0.05, **p < 0.01, ***p < 0.001.

## Discussion

Based on previous evidence that L2 reading speed may not be a reliable indicator of ease of processing, this study investigated the influence that individual differences in L2 knowledge and processing efficiency bear on online reading. In the present study, online processing measures were acquired by recording L2 learners’ eye movements during natural reading of Spanish sentences, which contained multiword units that were either congruent or non-congruent with their L1 (English). A recently developed measure of chunk sensitivity was employed as an index of processing efficiency in each of participants’ two languages. Overall, the data confirmed the expected L1-L2 congruency effect during processing of the target verbs of multiword units, replicating previous findings of costlier processing of L1-L2 incongruent multiword units, relative to congruent items. In this sense, the present study elaborated on previous work by showing that modified collocations, appearing as N-that-V relative noun phrases, yielded the same congruency effect reported in previous studies on V-N collocations.

However, the analysis revealed critical modulations of cross-linguistic influence at the individual level. At the group level, the results provided a clear replication of the L1 congruency effect reported for V-N phrases in which the verb is cross-linguistically incongruent (e.g., Wolter and Gyllstad, 2011, 2013). But the analysis of individual differences revealed that the effect was far from being homogeneously present in all readers. Rather, L1 and L2 chunk sensitivity measures, along with multiword-based proficiency, modulated processing costs between conditions as well as total reading speed. First, higher L1 chunk sensitivity was associated with faster reading times during early stages of processing, as captured by the measure of gaze duration. This finding was congruent with the only previously available study that examined the role of chunking ability in L2 processing (López-Beltrán et al., 2020). Secondly, the L2 measure of chunk sensitivity modulated performance in late stages of processing as indicated by total reading times. The analyses of total reading times of the critical verb region revealed an inverse U-shape effect, where the “poor chunkers” (low chunking-ability readers) who also had low L2 multiword-based proficiency showed some of the fastest reading times, but no significant differences between conditions, i.e., no congruency effect. This pattern indicated shallow processing, as can be gathered from the lower competence in the language and the less efficient processing of multiword chunks. On the other hand, individuals with high chunking ability and higher L2 multiword-based proficiency presented more efficient processing, with total faster reading times, as well as a significant difference between conditions. The intermediate values of the gamut presented a gradual slowdown in overall reading speed as knowledge of multiword units increased, but also a critical growth in between-condition differences as L2 chunking ability increased. Together, the results reveal joint contributions of knowledge of L2-specific multiword units and chunking ability, which modulate reading speed and processing efficiency. In what follows, the contribution of each factor is further discussed.

### Individual Chunking Ability Measures in the L1 and L2

One main goal of this study was to assess whether Chunk Sensitivity scores, either in the L1 or the L2, modulate reading times from L2 online processing measures (Research Question 1). The results indicated that individual scores of L1 Chunk Sensitivity were a significant predictor at early stages of L2 processing, as reflected by gaze duration. This is in line with the results reported by López-Beltrán et al. (2020), who found that L1 Chunk Sensitivity influenced self-paced reading times. It is relevant to note that gaze duration and self-paced reading both reflect the duration of a “first pass” in reading. In particular, because in self-paced paradigms the reader is not able to make regressive eye movements, this measure is *a priori* most comparable to gaze duration, which is computed based on the initial fixations on a region, before the eyes move on to the next word or move back to the left. The results reported here and in López-Beltrán and colleagues’ study appear to converge in showing that chunk sensitivity measured in the *native* language predicts efficiency in early stages of *non-native* language processing. The association between chunking ability measured in the native language and processing of a language system acquired later in life would suggest that efficiency in early lexical access is dependent on domain-general retrieval mechanisms that are best captured by the L1 chunk sensitivity measure.

A novel contribution in the present study is the finding that L2 chunking ability was predictive of later stages of (second) language processing. That is, while L1 Chunk Sensitivity tended to modulate early access in gaze duration times, the L2 Chunk Sensitivity scores modulated total duration times, which are believed to primarily affect integration and incremental processing. In particular, while gaze duration is believed to index “initial” processing (e.g., early lexical access), total reading times are thought of as associated with later stages of processing, i.e., post-lexical access and aggregate effects of incremental processing and integration in context (Rayner, 1998). Because once bottom-up information is accessed (e.g., in lexical access in early stages of processing) the information needs to be structured and integrated within its context, knowledge of language-specific combinatory rules would be critical to guide the later stages of processing. Indeed, if chunking (i.e., binding of different elements during processing) is dependent on the projection of long-term memory representations that guide the scaffolded incremental processing of discourse segments, then language-specific chunk sensitivity should be critical in modulating later stages of processing. The significant effect of L2 chunk sensitivity in total durations is congruent with this view. Together, the effects of L1 and L2 chunk sensitivity measures depict a time-course of bilingual processing, in which domain-general and language-specific skills play a role at different steps of the incremental process of extracting meaning out of input.

Therefore, I suggest that the differences between chunk sensitivity measures in the native and non-native language are associated with discrete aspects involved in binding and integrating chunks of information in the input. How exactly, then, do the measures of chunking ability differ when measured in the L1 or in the L2 of language learners? Because the chunking ability task measures sensitivity to familiar “chunks” in a given language, the scores obtained reflect both language experience and domain-general chunking ability. The language experience component, i.e., familiarity with frequently co-occurring chunks in the language, necessarily relies on an individual’s level of experience with a specific language. In this regard, an individual’s chunk sensitivity score will not be the identical in each of the languages measured. Nonetheless, the measure is also believed to go beyond the static level of familiarity with given “chunks”, and rather captures an individual’s ability to recruit knowledge from prior experience to bind individual elements together and to build associations online. The multifaceted nature of the measure is reflected in the fact that L1 and L2 Chunk Sensitivity scores were not significantly correlated in the lower-intermediate learners tested in the present study. The only available benchmark against which these results can be compared are those reported by López-Beltrán et al. (2020). In that study, the authors found a weak but significant correlation between the two versions of the task employed here (*r* = 0.37, *p* = 0.01). Although the exact same Chunk Sensitivity tasks were used in their study and the present one, an important difference is that the participants in the current study were recruited from basic Spanish courses (equivalent to the third and fourth semester), whereas López-Beltrán and colleagues tested students in upper-level and graduate Spanish courses. In accounting for the weak correlation between L1 and L2 chunk sensitivity, López-Beltrán and colleagues also speculated that L2 chunking ability may become a more reliable measure and more strongly correlated with L1 chunk sensitivity as L2 multiword knowledge increased. Their explanation is supported by the present dataset which indicates that, as they predicted, the association between chunk sensitivity in a L1 and L2 at lower proficiency levels is even weaker.

Finally, given the relative novelty of the Chunk Sensitivity measures, it is relevant to consider the validity of the tests, including aspects of predictive validity and construct validity. The extant evidence from L1 studies (McCauley and Christiansen, 2015; McCauley et al., 2017) and the first two L2 studies known to the author (i.e., this study and López-Beltrán et al.’s) has so far given robust support to the predictive validity of the tests. The dedicated analysis of subgroups (in the section “Follow-up analysis”) provided further indication that chunking ability specifically predicted online processing, while offline comprehension was predicted by the L2 proficiency measure. Additionally, an important question relative to construct validity is whether the Chunk Sensitivity measures employed here are independent from other known cognitive measures, such as PSTM or working memory (WM). With regard to PSTM, correlational tests in the current dataset revealed no significant correlation between PSTM and chunking ability in either language (English: *r* = 0.05, *p* = 0.76; Spanish: *r* = 0.07, *p* = 0.67). Additional relevant confirmation of construct validity comes from López-Beltrán and colleagues, who investigated the contributions of chunking ability (measured in English and Spanish) and WM (measured through the Operation-Span task) to online processing, by including these measures in their analysis. Their results confirmed that chunking ability was a significant predictor even after WM was included in the model; importantly, WM did not emerge as a significant predictor of online processing. That is, in both the present study and López-Beltrán et al.’s work, chunking ability was a significant predictor of online reading times, whereas neither WM nor PSTM emerged as significant predictors of online processing. Taken together, the extant evidence provides support to the independent contribution of chunking ability, and the validity of the Chunk Sensitivity measures employed here beyond the well-established measures of PSTM and WM.

### Chunking Ability Is a Modulator of Cross-Linguistic Influence in L2 Processing

Two additional goals were to investigate in what ways individual knowledge of L2-specific units predicts differences in online processing (RQ 2) and any potential interactions between L2 and chunking ability measures (RQ 3). The analysis took a different approach from previous studies that divided participants based on their overall reading speed (Hoover and Dwivedi, 1998; Roberts and Felser, 2011; Kaan et al., 2015). If both high- and low- efficient readers read fast, in a median split-based analysis, the individuals with the highest and lowest reading efficiency would be lumped together. In the absence of an adequate index of processing efficiency, previous studies found mixed evidence regarding the correlation of reading speed with proficiency measures (e.g., Roberts and Felser, 2011; Kaan et al., 2015). In the analysis reported here, total duration revealed a critical three-way interaction between L2 Chunk Sensitivity, L2 multiword knowledge and L1-L2 congruency in multiword units. Proficiency scores from L2 multiword knowledge –which tapped directly into the L2 aspect under consideration- were a significant predictor of gaze duration reading times.

A critical question was how chunking ability modulates cross-linguistic influences in processing (RQ 4). For incongruent multiword units, the results revealed the hypothesized inverse U-shaped pattern along the gradient of L2 chunk sensitivity, with faster reading times for individuals at both extremes of the chunking ability continuum (as illustrated in Figure 4). While, superficially, reading times may look similar in good and poor “chunkers,” the underlying causes are likely entirely different. That is, individuals with high chunking ability were believed to have more efficient online processing, in line with previous findings (McCauley and Christiansen, 2015; McCauley et al., 2017). On the other hand, poor chunking ability is associated with inefficient online computation of dependencies in the input, which may lead to faster (but shallow) reading. If this interpretation is correct, one would of course expect that shallow readers should be largely insensitive to the experimental manipulations and the properties of the input (i.e., cross-linguistic influence), while a more robust effect should be found in efficient processors. This prediction was confirmed by the analysis for readers at different levels of L2 multiword knowledge and chunking ability. Indeed, the results showed a lack of the well-attested L1-L2 congruency effect in the less advantaged readers. While individuals with poor L2 chunk sensitivity showed no significant effect of L1-L2 congruency, those with better chunking ability showed the effect. Remarkably, these patterns were not affected by L2 knowledge level. That is, the role of chunk sensitivity is highlighted by the finding that high chunking ability individuals showed the effect, regardless of L2 multiword knowledge, while the lower chunking ability readers showed a lack of congruency, even with higher L2 multiword knowledge. The results reported here provided evidence not only of shallow processing of information that requires L2-specific knowledge, but also showed that L2 chunk sensitivity can predict in what cases individual reading times will indicate shallow reading. In previous results reported by Kaan et al. (2015), the lack of the expected effects was interpreted as a clear indicator of shallow processing, but no correlates beyond the lack of differences between conditions were identified. The present results not only support this interpretation, but make a contribution by providing direct evidence of an association between L2 chunking ability and the modulation of L2 processing costs in different conditions. In short, both good and poor “chunkers” may read similarly fast, but for entirely different reasons.

Importantly, the present findings highlight that the way in which cross-linguistic influence emerges and is modulated by chunking ability may be non-linear. Clearly, the emergence of cross-linguistic differences requires a certain level of depth in processing; during shallow reading, in which the reader does not engage with L2-specific features in the input, expected effects of cross-linguistic influence may not be revealed. On the other end, with increasing chunking ability, the expected cross-linguistic influence is reduced as a consequence of more efficient processing. Future work will be needed to confirm this pattern by examining whether and how chunking ability modulates cross-linguistic differences, while also carefully controlling for individuals’ command of the specific L2 feature under examination. Testing how chunking ability impacts processing of phonological, morphosyntactic or lexical features will be important in further characterizing the scope of this modulator.

### How Generalizable Are Group-Based Findings? Implications for Research on L2 Processing

Based on the group averages, which replicated the well-attested L1-L2 congruency effect (e.g., Yamashita and Jiang, 2010; Wolter and Gyllstad, 2011, 2013; Carrol et al., 2016; Wolter and Yamashita, 2018), one could examine group-based grand averages, find the results satisfactory and in no need for further investigation. However, this would tell us only part of the story – and an imperfect one – about L2 processing and the impact of cross-linguistic influence. The results reported here evince that, without taking into consideration and appropriately accounting for individual differences, the congruency effect of the grand average is driven by about half of the learners in this sample. This would be, by all means, an inaccurate result and an inappropriate depiction of L2 processing in the tested population.

Given the critical importance of developing theories that are generalizable (i.e., extrapolated from a representative sample to the “real” population), reaching an accurate understanding of a given sample on which to ground our conclusions is imperative. The findings reported here provide evidence that group-based average reading times may provide a poor depiction of the individuals therein. As in previous L2 research, the results revealed substantial variability within the sample tested. By identifying chunking ability as a modulator of reading, the analysis identified systematic influences on reading patterns within the sample. The variability uncovered by the analyses highlights two striking points. First, the large amount of variability, with about half of the participants showing between-group differences but with no significant effect in about half of the sample, underscores the coarseness of the group-based measure. Secondly, given the independent contribution of chunking ability, the fact that this and previous studies have reliably replicated a congruency effect is in itself remarkable. The important point is that strong effects may be replicable even if they are far less frequent in the general population. To illustrate, based on the present findings, one could expect the congruency effect to be reliably found at the group level (given a large enough sample) but, at the level of the individual learner, it could be expected to be found in about half of the cases. Therefore, it is not only important to control for potential idiosyncracies present within a given sample, but also to understand and account for the existence of variety within the group^10^.

In summary, the findings reported here call for a renewed attempt in processing studies to characterize individual variability within groups of participants. Provided sufficiently large sample sizes, the approach presented here integrating measures of chunking ability is an invitation to reanalyze datasets and to, potentially, better characterize the performance of subgroups of individuals within the sample. While efforts to explore the role of chunking ability are just beginning, the extant evidence already suggests that its influence affects L2 processing of aspects as varied as cross-language congruency in multiword units (in the present results) and morphosyntactic expression of gender (López-Beltrán et al., 2020). Future studies should investigate how the clear association between multiword chunking ability and processing explored here will hold when other linguistic aspects are examined, e.g., in L2 syntax or morphosyntax; future work should also pursue conceptual replications using different sets of multiword units and including also comparisons to L1 speakers. Large-scale studies as well as work in other languages will be instrumental to further validate chunking-based measures; for instance, interesting questions arise regarding chunking in languages with different writing systems. The data available make a case for the consideration of chunking ability as a critical modulator of individual differences in L2 reading, and in language processing more generally.

## Conclusion

This article addresses a gap in previous studies which reported signs of shallow processing during L2 reading, but found that neither language proficiency nor reading speed alone were reliable predictors of online reading performance. Instead, the results in this study identified chunking ability as a critical modulator of online L2 processing and of cross-linguistic influences in particular. Crucially, the data highlight the fact that only a fraction of the fastest reading speeds can be attributed to high processing efficiency (i.e., chunk sensitivity) or to higher L2 experience, while many other fast reading times are directly associated with poor chunking ability. Altogether, the findings suggest that incorporating measures of chunking ability in the L1 and L2 might add a fundamental dimension to account for processing effects, not only at the level of the individual but also in the aggregate – i.e., at the group level. Future replication studies, including potential work on more typologically distant languages, will be required to confirm the role of L1 and L2 chunking ability. The present results are particularly promising given that the modulators of L2 reading efficiency have proven to be an elusive target in previous work. Analyses that miss this modulator may be unable to capture processing efficiency as a true source of variability. In conclusion, the findings have potentially far-reaching implications for the interpretation of previous and future results derived from online reading times, and invite future work to explore the contribution of individual chunking ability by factoring it into investigations of L2 online processing.

## Data Availability Statement

The raw data supporting the conclusions of this article will be made available by the authors, without undue reservation.

## Ethics Statement

The studies involving human participants were reviewed and approved by the Institutional Review Board, Penn State University. The participants provided their written informed consent to participate in this study.

## Author Contributions

The author confirms being the sole contributor of this work and has approved it for publication.

## Conflict of Interest

The author declares that the research was conducted in the absence of any commercial or financial relationships that could be construed as a potential conflict of interest.

## Footnotes

1 Importantly, this fact is still fully coherent with the eye-mind assumption, in that less time spent looking at a word still indicates less time processing that word, even if the underlying cause is, e.g., lack of engagement.

2 An “observed power” estimation performed with the *simr* package in R (v. 1.0, Green and MacLeod, 2016) indicated that a sample of *N* = 25 was sufficient to reach 80% power, alpha = 0.05.

3 An additional advantage of PMI is its computational simplicity, which allows to develop semantic relatedness measures for more comprehensive lists of words, including words without very high frequencies.

4 In English the “that” complementizer is optional; it is always expressed in Spanish, making the relative structure explicit. Besides other structural differences (i.e., English requires overt subjects, but these are optional in Spanish) sentence have equivalent syntax across languages, as in the examples in Table 1.

5 While the individual word test employed here is highly relatable to the materials employed, it may also be limited in terms of its generalizability and characterization of participants’ overall level of proficiency. To overcome this limitation, future work should incorporate other normed tests (e.g., the Spanish version of the Lextale test; Izura et al., 2014).

6 One item displayed an erroneous carrier sentence; excluding this item did not affect the results of the analyses.

7 Measures of data skewness revealed that total duration times were right-skewed (skewness for total duration of noun region: 1.50 and the verb region: 1.60); after being log-normalized, skewness was corrected (noun region: −0.10; verb region: 0.11). The gaze duration data was not right-skewed, i.e., unlike total reading times, they did not contain small numbers of observations with very high reading time values (noun region: 0.14; verb region: 0.37). Because applying a log transformation was not necessary to correct skewness and resulted in artificially non-normally distributed gaze duration data (skewness for noun region: −2.82; verb region: −2.08), gaze duration was not log-transformed.

8 A tercile split follows the same procedure as a median split, but the sample is divided into three quantiles instead of two (lower, medium and upper).

9 I am grateful for the suggestion from one of the reviewers to analyze the preamble to address this point. Although the preamble sentences were not controlled or manipulated with any particular aspect of the L2 in mind, they were identical for all participants and therefore provide an additional opportunity to peruse the predictive power of individual Chunk Sensitivity during reading of the L2 more generally.

10 Additional factors may also influence individuals differently, e.g., it may also be that the reversed order of collocations in N-that-V phrases posed greater demands on processing than canonical V-N phrases would, perhaps affecting particularly individuals with low chunking ability. Relatedly, individuals may show different sensitivity to other stimulus features (e.g., incongruent collocations tend to be less transparent; Yamashita, 2018). More research will be needed to further investigate how features such as the degree of familiarity or transparency may interact with chunking ability.

## References

[B1] Alderson J. C. (1984). “Reading in a foreign language: a reading problem or a language problem?,” in *Reading in a Foreign Language*, eds Alderson J. C. Urquhart A. H. (London: Longman), 1–24.

[B2] Alderson J. C. (1993). “The relationship between grammar and reading in an English for academic purposes test battery,” in *A New Decade of Language Testing Research: Selected Papers from the 1990 Language Testing Research Colloquium: Dedicated in Memory of Michael Canale*, eds Douglas D. Chapelle C. (Alexandria, VA: Teachers of English to Speakers of Other Languages), 203–219.

[B3] Baddeley A. Papagno C. Vallar G. (1988). When long-term learning depends on short-term storage. *J. Mem. Lang.* 27 586–595. 10.1016/0749-596X(88)90028-9

[B4] Baddeley A. D. (1979). *Working Memory and Reading. In Processing of Visible Language.* Boston, MA: Springer, 355–370.

[B5] Barr D. J. Levy R. Scheepers C. Tily H. J. (2013). Random effects structure for confirmatory hypothesis testing: keep it maximal. *J. Mem. Lang.* 68 255–278. 10.1016/j.jml.2012.11.001 24403724 PMC3881361

[B6] Bates D. Maechler M. Bolker B. Walker S. (2015). Fitting linear mixed-effects models using lme4. *J. Stat. Softw.* 67 1–48.

[B7] Bowey J. A. (1986). Syntactic awareness in relation to reading skill and ongoing reading comprehension monitoring. *J. Exp. Child Psychol.* 41 282–299. 10.1016/0022-0965(86)90041-x

[B8] Broughton S. H. Sinatra G. M. Reynolds R. E. (2010). The nature of the refutation text effect: an investigation of attention allocation. *J. Educ. Res.* 103 407–423. 10.1080/00220670903383101

[B9] Budiu R. Royer C. Pirolli P. L. (2007). “Modeling information scent: a comparison of LSA, PMI and GLSA similarity measures on common tests and corpora,” in *Proceedings of the 8th Annual Conference of the Recherche d’Information Assistée par Ordinateur (RIAO)*, (Pittsburgh, PA: Centre des Hautes Études Internationales d’Informatique Documentaire).

[B10] Bullinaria J. A. Levy J. P. (2007). Extracting semantic representations from word co-occurrence statistics: a computational study. *Behav. Res. Methods* 39 510–526. 10.3758/bf03193020 17958162

[B11] Carrol G. Conklin K. (2017). Cross language lexical priming extends to formulaic units: evidence from eye-tracking suggests that this idea “has legs.”. *Biling. Lang. Cogn.* 20 299–317. 10.1017/S1366728915000103

[B12] Carrol G. Conklin K. Gyllstad H. (2016). Found in translation: the influence of the L1 on the reading of idioms in a L2. *Stud. Second Lang. Acquis.* 38 403–433. 10.1017/S0272263115000492

[B13] Christiansen M. H. Chater N. (2016). The Now-or-Never bottleneck: a fundamental constraint on language. *Behav. Brain Sci.* 39 1–72. 10.1017/S0140525X1500031X 25869618

[B14] Cieri C. David M. Kevin W. (2004). “The fisher corpus: a resource for the next generations of speech-to-text,” in *Proceedings of the 4th International Conference on Language Resources and Evaluation*, (Lisbon). 10.1007/978-94-017-1183-8_1

[B15] Clahsen H. Felser C. (2006). Grammatical processing in language learners. *Appl. Psycholinguist.* 27 3–42. 10.1017/s0142716406060024 19337839

[B16] Coughlin C. E. Tremblay A. (2012). Proficiency and working memory based explanations for nonnative speakers’ sensitivity to agreement in sentence processing. *Appl. Psycholinguist.* 34 615–646. 10.1017/S0142716411000890

[B17] Daneman M. Carpenter P. A. (1980). Individual differences in working memory and reading. *J. Mem. Lang.* 19 450–466.

[B18] Davies M. (2016). *Corpus del Español: Two billion words, 21 Countries.* Available online at http://www.corpusdelespanol.org/web-dial/ (accessed October 15, 2018).

[B19] Dussias P. E. (2010). Uses of eye-tracking data in second language sentence processing research. *Annu. Rev. Appl. Linguist.* 30 149–166. 10.1017/s026719051000005x

[B20] Elliott L. L. (1962). Backward and forward masking of probe tones of different frequencies. *J. Acoust. Soc. Am.* 34 1116–1117. 10.1121/1.1918254

[B21] Erçetin G. Alptekin C. (2013). The explicit/implicit knowledge distinction and working memory: implications for second-language reading comprehension. *Appl. Psycholinguist.* 34 727–753. 10.1017/s0142716411000932

[B22] Felser C. Roberts L. Marinis T. Gross R. (2003). The processing of ambiguous sentences by first and second language learners of English. *Appl. Psycholinguist.* 24 453–489. 10.1017/s0142716403000237

[B23] Ferreira F. (2003). The misinterpretation of noncanonical sentences Cognitive Psychology. *Cogn. Psychol.* 47 164–203. 10.1016/S0010-0285(03)00005-7 12948517

[B24] Ferreira F. Christianson K. Hollingworth A. (2001). Misinterpretations of garden-path sentences: implications for models of sentence processing and reanalysis. *J. Psycholinguist. Res.* 30 3–20. 11291182 10.1023/a:1005290706460

[B25] Ferreira F. Patson N. D. (2007). The?Good Enough? approach to language comprehension. *Lang. Linguist. Compass* 1 71–83. 10.1111/j.1749-818x.2007.00007.x

[B26] Foucart A. Frenck-Mestre C. (2011). Grammatical gender processing in L2: electrophysiological evidence of the effect of L1-L2 syntactic similarity. *Bilingualism* 14 379–399. 10.1017/S136672891000012X

[B27] Fox J. Friendly G. G. Graves S. Heiberger R. Monette G. Nilsson H. (2007). *The Car Package.* Boston, MA: R Foundation for Statistical Computing.

[B28] Frenck-Mestre C. (2005). Eye-movement recording as a tool for studying syntactic processing in a second language: a review of methodologies and experimental findings. *Second Lang. Res.* 21 175–198. 10.1191/0267658305sr257oa

[B29] Gathercole S. E. Pickering S. J. Hall M. Peaker S. M. (2001). Dissociable lexical and phonological influences on serial recognition and serial recall. *Q. J. Exp. Psychol. Section A* 54 1–30. 10.1080/02724980042000002 11216312

[B30] Green P. MacLeod C. J. (2016). SIMR: an R package for power analysis of generalized linear mixed models by simulation. *Methods Ecol. Evol.* 7 493–498. 10.1111/2041-210X.12504

[B31] Harrington M. Sawyer M. (1992). L2 working memory capacity and L2 reading skill. *Stud. Second Lang. Acquisit.* 14 25–38. 10.1017/s0272263100010457

[B32] Havik E. Roberts L. Van Hout R. Schreuder R. Haverkort M. (2009). Processing subject–object ambiguities in the L2: A self-paced reading study with German L2 learners of Dutch. *Lang. Learn* 59 73–112. 10.1111/j.1467-9922.2009.00501.x

[B33] Hoover M. Dwivedi V. (1998). Syntactic processing by skilled bilinguals. *Lang. Learn.* 48 1–29. 10.1111/1467-9922.00031

[B34] Hopp H. (2006). Syntactic features and reanalysis in near-native processing. *Second Lang. Res.* 22 369–397. 10.1191/0267658306sr272oa

[B35] Hopp H. (2010). Ultimate attainment in L2 inflection: performance similarities between non-native and native speakers. *Lingua* 120 901–931. 10.1016/j.lingua.2009.06.004

[B36] Hopp H. (2013). Grammatical gender in adult L2 acquisition: relations between lexical and syntactic variability. *Second Lang. Res.* 29 33–56. 10.1177/0267658312461803

[B37] Hopp H. (2014). Working memory effects in the L2 processing of ambiguous relative clauses. *Lang. Acquisit.* 21 250–278. 10.1080/10489223.2014.892943

[B38] Hopp H. (2015). The timing of lexical and syntactic processes in L2 sentence comprehension. *Appl. Psycholinguist.* 39 1–28.

[B39] Hsu N. S. Novick J. M. (2016). Dynamic engagement of cognitive control modulates recovery from misinterpretation during real-time language processing. *Psychol. Sci.* 27 572–582. 10.1177/0956797615625223 26957521 PMC4833548

[B40] Izura C. Cuetos F. Brysbaert M. (2014). Lextale-Esp: a test to rapidly and efficiently assess the Spanish vocabulary size. *Psicológica* 35 49–66.

[B41] Jeon E. H. Yamashita J. (2014). L2 reading comprehension and its correlates: a meta-analysis. *Lang. Learn.* 64 160–212. 10.1111/lang.12034

[B42] Jiang N. (2015). “Six decades of research on bilingual presenattion,” in *The Cambridge Handbook of Bilingual Processing*, ed. Schwieter J. W. (Cambridge: Cambridge University Press), 29–84. 10.1017/cbo9781107447257.002

[B43] Juffs A. (2004). Representation, processing and working memory in a second language. *Trans. Philol. Soc.* 102 199–225. 10.1111/j.0079-1636.2004.00135.x

[B44] Just M. A. Carpenter P. A. (1980). “A theory of reading: from eye fixations to comprehension,” in *Psychological Review*, ed. Holyoak K. J. (Washington, D.C: American Psychological Association), 87. 7413885

[B45] Kaan E. Ballantyne J. C. Wijnen F. (2015). Effects of reading speed on second-language sentence processing. *Appl. Psycholinguist.* 36 799–830. 10.1017/S0142716413000519

[B46] Karimi H. Ferreira F. (2016). Good-enough linguistic representations and online cognitive equilibrium in language processing. *Q. J. Exp. Psychol.* 69 1013–1040. 10.1080/17470218.2015.1053951 26103207

[B47] Keating G. D. Jegerski J. (2015). Experimental designs in sentence processing research: a methodological review and user’s guide. *Stud. Second Lang. Acquisit.* 37 1–32. 10.1017/s0272263114000187

[B48] Kieffer M. J. Lesaux N. K. (2008). The role of derivational morphology in the reading comprehension of Spanish-speaking English language learners. *Read. Writ. Interdiscip. J.* 21 783–804. 10.1007/s11145-007-9092-8

[B49] Kieffer M. J. Lesaux N. K. (2012a). Direct and indirect roles of morphological awareness in the English reading comprehension of native Spanish, Filipino, Vietnamese, and English speakers. *Lang. Learn.* 62 1170–1204. 10.1111/j.1467-9922.2012.00722.x

[B50] Kieffer M. J. Lesaux N. K. (2012b). Knowledge of words, knowledge about words: dimensions of vocabulary in first and second language learners in sixth grade. *Read. Writ.* 25 347–373. 10.1007/s11145-010-9272-9

[B51] Koda K. (2005). *Insights into Second Language Reading: A Cross-Linguistic Approach.* New York, NY: Cambridge University Press.

[B52] Kotz S. A. Elston-Güttler K. (2004). The role of proficiency on processing categorical and associative information in the L2 as revealed by reaction times and event-related brain potentials. *J. Neurolinguist.* 17 215–235. 10.1016/S0911-6044(03)00058-7

[B53] Kyriacou M. Conklin K. Thompson D. (2020). Passivizability of idioms: has the wrong tree been barked up? *Lang. Speech* 63 404–435. 10.1177/0023830919847691 31106699

[B54] Laufer B. Girsai N. (2008). Form-focused instruction in second language vocabulary learning: a case for contrastive analysis and translation. *Appl. Linguist.* 29 694–716. 10.1093/applin/amn018

[B55] Leys C. Ley C. Klein O. Bernard P. Licata L. (2013). Detecting outliers: do not use standard deviation around the mean, use absolute deviation around the median. *J. Exp. Soc. Psychol.* 49 764–766. 10.1016/j.jesp.2013.03.013

[B56] Long J. A. (2019). *Interactions**: Comprehensive, User-Friendly Toolkit for Probing Interactions. R package version 1.1.0.* Available online at: https://cran.r-project.org/package=interactions (accessed July 20, 2020).

[B57] López-Beltrán P. Pulido M. F. Dussias P. E. Christiansen M. (2020). *Individual Differences in Chunking Ability Predict Native-Like Second Language Processing: Evidence from Spanish Gender Agreement. Submitted for Publication.* Available online at: https://osf.io/vx5g8/?view_only=48ba0e2fdba14ad4b182ac9f90cfea13 (accessed July 20, 2020).

[B58] Marian V. Blumenfeld H. K. Kaushanskaya M. (2007). The language experience and proficiency questionnaire (LEAP-Q): assessing language profiles in bilinguals and multilinguals. *J. Speech Lang. Hear. Res.* 50 940–967. 10.1044/1092-4388(2007/067) 17675598

[B59] Marsden E. Thompson S. Plonsky L. (2018). A methodological synthesis of self-paced reading in second language research. *Appl. Psycholinguist.* 39 861–904. 10.1017/s0142716418000036

[B60] Martin K. I. Ellis N. C. (2012). The roles of phonological short-term memory and working memory in L2 grammar and vocabulary learning. *Stud. Second Lang. Acquisit.* 34 379–413. 10.1017/S0272263112000125

[B61] McCauley S. M. Christiansen M. H. (2015). “Individual differences in chunking ability predict on-line sentence processing individual differences in chunking ability predict on-line sentence processing,” in *Proceedings of the 37th Annual Conference of the Cognitive Science Society*, (Pasadena, CA), 1553–1558.

[B62] McCauley S. M. Isbilen E. S. Christiansen M. H. (2017). “Chunking ability shapes sentence processing at multiple levels of abstraction,” in *Proceedings of the 39th Annual Conference of the Cognitive Science Society*, eds Gunzelmann G. Howes A. Tenbrink T. Davelaar E. J. (Austin, TX: Cognitive Science Society), 2681–2686. Available online at: http://cnl.psych.cornell.edu/pubs/2017-mic-cogsci.pdf

[B63] Metzner P. von der Malsburg T. Vasishth S. Rösler F. (2017). The importance of reading naturally: evidence from combined recordings of eye movements and electric brain potentials. *Cogn. Sci.* 41 1232–1263. 10.1111/cogs.12384 27307404

[B64] Miller B. W. (2015). Educational psychologist using reading times and eye-movements to measure cognitive engagement. *Educ. Psychol.* 50 31–42. 10.1080/00461520.2015.1004068

[B65] Miller G. A. Taylor W. G. (1948). The perception of repeated bursts of noise. *J. Acoust. Soc. Am.* 20 171–182. 10.1121/1.1906360

[B66] Navarro-Torres C. A. Garcia D. L. Chidambaram V. Kroll J. F. (2019). Cognitive control facilitates attentional disengagement during second language comprehension. *Brain Sci.* 9 19–21. 10.3390/brainsci9050095 31035554 PMC6562798

[B67] Nesselhauf N. (2003). The use of collocations by advanced learners of English. *Appl. Linguist.* 24 223–242. 10.1093/applin/24.2.223

[B68] Novick J. M. Hussey E. Teubner-Rhodes S. Harbison J. I. Bunting M. F. (2013). Clearing the garden-path: improving sentence processing through cognitive control training. *Lang. Cogn. Neurosci.* 29 186–217. 10.1080/01690965.2012.758297

[B69] Pashler H. (1988). Familiarity and visual change detection. *Percept. Psychophys.* 44 369–378. 10.3758/bf03210419 3226885

[B70] Payne T. W. Kalibatseva Z. Jungers M. K. (2009). Does domain experience compensate for working memory capacity in second language reading comprehension? *Learn. Individ. Differ.* 19 119–123. 10.1016/j.lindif.2008.05.003

[B71] Pulido M. F. (2020). Native language inhibition predicts more successful second language learning: evidence of two ERP pathways during learning. *Neuropsychologia* 10.1016/j.neuropsychologia.2020.107732 [Epub ahead of print]. 33347916

[B72] Pulido M. F. Dussias P. E. (2020). Desirable difficulties while learning collocations in a second language: conditions that induce L1 interference improve learning. *Biling. Lang. Cogn.* 23 652–667. 10.1017/S1366728919000622

[B73] Rayner K. (1998). Eye movements in reading and information processing: 20 years of research. *Psychol. Bull.* 124 372–422. 10.1037/0033-2909.124.3.372 9849112

[B74] Recchia G. Jones M. N. (2009). More data trumps smarter algorithms: comparing pointwise mutual information with latent semantic analysis. *Behav. Res. Methods* 41 647–656. 10.3758/BRM.41.3.647 19587174

[B75] Remez R. E. Ferro D. F. Dubowski K. R. Meer J. Broder R. S. Davids M. L. (2010). Is desynchrony tolerance adaptable in the perceptual organization of speech? *Atten. Percept. Psychophys.* 72 2054–2058. 10.3758/bf03196682 21097850

[B76] Reppen R. Ide N. Suderman K. (2005). *American National Corpus. Philadelphia: Linguistic Data Consortium.* Available online at: http://americannationalcorpus.org/

[B77] Roberts L. (2012). Individual differences in second language sentence processing. *Lang. Learn.* 62 172–188. 10.1111/j.1467-9922.2012.00711.x

[B78] Roberts L. Felser C. (2011). Plausibility and recovery from garden paths in second language sentence processing. *Appl. Psycholinguist.* 32 299–331. 10.1017/S0142716410000421

[B79] Sagarra N. Herschensohn J. (2010). The role of proficiency and working memory in gender and number agreement processing in L1 and L2 Spanish. *Lingua* 120 2022–2039. 10.1016/j.lingua.2010.02.004

[B80] Shiotsu T. (2010). *Components of L2 Reading: Linguistic and Processing Factors in The Reading Test Performances of Japanese EFL Learners.* New York, NY: Cambridge University Press.

[B81] Siegel L. S. (1994). Working memory and reading: a life-span perspective. *Int. J. Behav. Dev.* 17 109–124. 10.1177/016502549401700107

[B82] Siyanova-Chanturia A. Conklin K. Schmitt N. (2011a). Adding more fuel to the fire: an eye-tracking study of idiom processing by native and non-native speakers. *Second Lang. Res.* 27 251–272. 10.1177/0267658310382068

[B83] Siyanova-Chanturia A. Conklin K. van Heuven W. J. B. (2011b). Seeing a Phrase “Time and Again” Matters: the role of phrasal frequency in the processing of multiword sequences. *J. Exp. Psychol. Learn. Mem. Cogn.* 37 776–784. 10.1037/a0022531 21355667

[B84] Streiner D. L. (2003). Starting at the beginning: an introduction to coefficient alpha and internal consistency. *J. Pers. Assess.* 80 99–103. 10.1207/S15327752JPA8001_18 12584072

[B85] Tavakol M. Dennick R. (2011). Making sense of Cronbach’s alpha. *Int. J. Med. Educ.* 2 53–55. 10.5116/ijme.4dfb.8dfd 28029643 PMC4205511

[B86] Tokowicz N. MacWhinney B. (2005). Implicit and explicit measures of sensitivity to violations in second language grammar. *Stud. Second Lang. Acquisit.* 27 173–204.

[B87] Vilkaitė L. (2016). Are nonadjacent collocations processed faster? *J. Exp. Psychol. Learn. Mem. Cogn.* 42:1632. 10.1037/xlm0000259 26913935

[B88] Vilkaite L. Schmitt N. (2019). Reading collocations in an L2:Do collocation processing benefits extend to non-adjacent collocations? *Appl. Linguist.* 40 329–354. 10.1093/applin/amx030

[B89] Wolter B. Gyllstad H. (2011). Collocational links in the L2 mental lexicon and the influence of l1 intralexical knowledge. *Appl. Linguist.* 32 430–449. 10.1093/applin/amr011

[B90] Wolter B. Gyllstad H. (2013). Frequency of input and L2 collocational processing. *Stud. Second Lang. Acquisit.* 35 451–482. 10.1017/S0272263113000107

[B91] Wolter B. Yamashita J. (2018). Word frequency, collocational frequency, L1 congruency, and proficiency in L2 collocational processing: what accounts for L2 performance? *Stud. Second Lang. Acquisit.* 40 395–416. 10.1017/S0272263117000237

[B92] Yamashita J. (2018). Possibility of semantic involvement in the L1-L2 congruency effect in the processing of L2 collocations. *J. Second Lang. Stud.* 1 60–78. 10.1075/jsls.17024.yam

[B93] Yamashita J. Jiang N. (2010). L1 influence on the acquisition of L2 collocations: Japanese ESL users and EFL learners acquiring English collocations. *TESOL Q.* 44 647–668. 10.5054/tq.2010.235998 24853049

