# Supplementary material for: Individual Chunking Ability Predicts Efficient or Shallow L2 Processing: Eye-Tracking Evidence From Multiword Units in Relative Clauses
Source: Front Psychol. 2021 Jan 15;11:607621. doi: 10.3389/fpsyg.2020.607621 (PMC7844092; doi:10.3389/fpsyg.2020.607621)
Supplement: Supplementary file 1 [file Table_1.docx]

**Multiword units test**

In the multiword units test, L1 English collocations were presented on the screen, one at a time. Participants were asked to type at their own pace the appropriate Spanish equivalent that had been previously learned and practiced (see Pulido, 2020 for learning and practice procedures). The test provided a measure of multiword proficiency by requiring learners to recall both meaning and form. Typed responses were coded for accuracy. Misspellings were not penalized as long as no more than two phonemes were incorrect, and the response could be interpreted unambiguously.

*List of stimuli (L1-L2 incongruent collocations)*

|  |  | | |  | | |  | | |
| --- | --- | --- | --- | --- | --- | --- | --- | --- | --- |
|  | verb | (det.) | noun |  | verb | (det.) | | noun |  |
| 1 | *pedir* ‘*ask for’* |  | *pizza* | 15 | *marcar ‘mark’* | [*un*] | | *número* |  |
|  | order |  | pizza |  | dial | [a] | | número |  |
| 2 | *dirigir ‘direct’* | [*un*] | *negocio* | 16 | *hacer ‘make’* | [*las*] | | *maletas* |  |
|  | run | [a] | business |  | pack | [the] | | bags |  |
| 3 | *rodar ‘roll’* |  | *escenas* | 17 | *controlar ‘control’* | [*el*] | | *estrés* |  |
|  | shoot |  | *scenes* |  | handle | [the] | | stress |  |
| 4 | *blanquear ‘whiten’* |  | *dinero* | 18 | *volar ‘fly’* |  | | *puentes* |  |
|  | launder |  | money |  | blow up |  | | *bridges* |  |
| 5 | *despertar ‘awaken’* |  | *dudas* | 19 | *navegar ‘sail’* | [*la*] | | *web* |  |
|  | raise |  | doubts |  | surf | [the] | | web |  |
| 6 | *poner ‘put’* |  | *atención* | 20 | *reconocer ‘recognize’* |  | | *fallos* |  |
|  | pay |  | attention |  | acknowledge |  | | mistakes |  |
| 7 | *dar ‘give’* |  | *paseos* | 21 | *presenciar ‘be present’* | | | *muertes* |  |
|  | take |  | walks |  | witness |  | | deaths |  |
| 8 | *perder ‘lose’* | [*un*] | *tren* | 22 | *entregar ‘deliver’* |  | | *propuestas* |  |
|  | miss | [a] | train |  | submit |  | | proposals |  |
| 9 | *ganar ‘win’* |  | *tiempo* | 23 | *programar ‘program’* | | | *citas* |  |
|  | buy |  | time |  | schedule |  | | appointments |  |
| 10 | *gastar ‘spend’* |  | *bromas* | 24 | *echar ‘throw’* |  | | *agua* |  |
|  | play |  | jokes |  | pour |  | | water |  |
| 11 | *abrir ‘open’* | [*el*] | camino | 25 | *cambiar ‘change’* |  | | *cheques* |  |
|  | lead | [the] | way |  | cash |  | | checks |  |
| 12 | *montar ‘assemble’* |  | *fiestas* | 26 | *subir ‘take up’* |  | | *documentos* |  |
|  | throw |  | parties |  | upload |  | | documents |  |
| 13 | *sacar* ‘extract’ |  | *fuerzas* | 27 | *publicar ‘publish’* | | | *vídeos* |  |
|  | draw |  | strength |  | post |  | | videos |  |
| 14 | *revelar ‘reveal’* |  | *fotos* | 28 | *ajustar ‘adjust’* |  | | *cuentas* |  |
|  | develop |  | photos |  | settle |  | | accounts |  |

*Note.* The table presents the collocations (i.e., conventional verb-noun phrases) that were studied and practiced by learners in Pulido (2020). Below each Spanish collocation, its idiomatic English translation is provided. The literal English translations for the verbs Spanish collocations are provided to the right of each verb.

Pulido, M. F. (2020). Native language inhibition predicts more successful second language learning: Evidence of two ERP pathways during learning. *Neuropsychologia.* Advance online publication. <https://doi.org/10.1016/j.neuropsychologia.2020.107732>
