# Supplementary material for: Individual Chunking Ability Predicts Efficient or Shallow L2 Processing: Eye-Tracking Evidence From Multiword Units in Relative Clauses
Source: Front Psychol. 2021 Jan 15;11:607621. doi: 10.3389/fpsyg.2020.607621 (PMC7844092; doi:10.3389/fpsyg.2020.607621)
Supplement: Supplementary file 3 [file Table_3.DOCX]

Individual Words Test

Start of Block: Default Question Block

Q2 Date (month/day/year)

________________________________________________________________

Q3 Participant code

________________________________________________________________

Q1 Welcome to this vocabulary test. 


In this test you will see a Spanish word and multiple words in English. Please select the option that is a correct translation.

End of Block: Default Question Block

Start of Block: verbs

Q4 pedir

- to ask for (1)
- to run (2)
- to pay (3)
- to try (4)

Q5 dirigir

- to play (1)
- to appeal (2)
- to charge (3)
- to direct (4)

Q6 rodar

- to hang (1)
- to roll (2)
- to blow (3)
- to tuck (4)

Q7 volar

- to map (1)
- to pop (2)
- to fly (3)
- to gun (4)

Q8 despertar

- to wake (1)
- to dut (2)
- to hunt (3)
- to ring (4)

Q9 poner

- to own (1)
- to put (2)
- to use (3)
- to end (4)

Q10 dar

- to look (1)
- to part (2)
- to give (3)
- to come (4)

Q11 perder

- to warm (1)
- to tend (2)
- to pick (3)
- to lose (4)

Q12 ganar

- to win (1)
- to hit (2)
- to box (3)
- to sit (4)

Q13 gastar

- to view (1)
- to spend (2)
- to cross (3)
- to draw (4)

Q14 abrir

- to form (1)
- to tell (2)
- to open (3)
- to hold (4)

Q15 montar

- to fail (1)
- to aid (2)
- to roll (3)
- to ride (4)

Q16 sacar

- to extract (1)
- to adapt (2)
- to lift (3)
- to wish (4)

Q17 revelar

- to stay (1)
- to reveal (2)
- to risk (3)
- to fall (4)

Q18 marcar

- to hold (1)
- to list (2)
- to mark (3)
- to miss (4)

Q19 hacer

- to be (1)
- to back (2)
- to come (3)
- to make (4)

Q20 controlar

- to control (1)
- to support (2)
- to work (3)
- to present (4)

Q21 publicar

- to exclude (1)
- to publish (2)
- to lend (3)
- to forgive (4)

Q22 navegar

- to owe (1)
- to tie (2)
- to sail (3)
- to tune (4)

Q23 reconocer

- to recover (1)
- to install (2)
- to search (3)
- to recognize (4)

Q24 presenciar

- to be present (1)
- to support (2)
- to work (3)
- to provide (4)

Q25 entregar

- to avoid (1)
- to deliver (2)
- to succeed (3)
- to realize (4)

Q26 fijar

- to visit (1)
- to send (2)
- to fix (3)
- to win (4)

Q27 echar

- to arise (1)
- to frame (2)
- to feel (3)
- to throw (4)

Q28 cambiar

- to change (1)
- to have (2)
- to look (3)
- to become (4)

Q29 subir

- to fish (1)
- to rise (2)
- to fall (3)
- to lack (4)

Q30 blanquear

- to align (1)
- to adjoin (2)
- to whiten (3)
- to append (4)

Q31 ajustar

- to retire (1)
- to guide (2)
- to pilot (3)
- to adjust (4)

Q32 ordenar

- to order (1)
- to state (2)
- to water (3)
- to ask (4)

Q33 correr

- to let (1)
- to run (2)
- to cut (3)
- to try (4)

Q34 disparar

- to relax (1)
- to hurry (2)
- to shoot (3)
- to rank (4)

Q35 lavar

- to hang (1)
- to roll (2)
- to feed (3)
- to wash (4)

Q36 levantar

- to raise (1)
- to enjoy (2)
- to fund (3)
- to track (4)

Q37 pagar

- to run (1)
- to pay (2)
- to ask (3)
- to try (4)

Q38 tomar

- to work (1)
- to make (2)
- to take (3)
- to come (4)

Q39 extrañar

- to stay (1)
- to risk (2)
- to wish (3)
- to miss (4)

Q40 comprar

- to buy (1)
- to win (2)
- to sit (3)
- to hit (4)

Q41 jugar

- to give (1)
- to play (2)
- to land (3)
- to move (4)

Q42 liderar

- to plan (1)
- to stop (2)
- to lead (3)
- to meet (4)

Q43 tirar

- to fear (1)
- to stick (2)
- to feel (3)
- to throw (4)

Q44 dibujar

- to draw (1)
- to pass (2)
- to save (3)
- to vote (4)

Q45 desarrollar

- to sit (1)
- to develop (2)
- to create (3)
- to claim (4)

End of Block: verbs

Start of Block: nouns

Q46 pizza

- greed (1)
- boxer (2)
- pizza (3)
- fold (4)

Q47 negocio

- interest (1)
- problem (2)
- question (3)
- business (4)

Q48 escenas

- scenes (1)
- fight (2)
- judge (3)
- user (4)

Q49 puentes

- leaves (1)
- bridges (2)
- coffees (3)
- factors (4)

Q50 interés

- business (1)
- position (2)
- interest (3)
- evidence (4)

Q51 atención

- situation (1)
- president (2)
- behavior (3)
- attention (4)

Q52 paseos

- walks (1)
- shops (2)
- notes (3)
- sales (4)

Q53 tren

- tree (1)
- train (2)
- stone (3)
- grant (4)

Q54 tiempo

- power (1)
- means (2)
- time (3)
- level (4)

Q55 bromas

- tools (1)
- bags (2)
- tales (3)
- jokes (4)

Q56 camino

- way (1)
- club (2)
- king (3)
- news (4)

Q57 fiestas

- capital (1)
- parties (2)
- manager (3)
- produce (4)

Q58 fuerzas

- relation (1)
- solution (2)
- strength (3)
- subjects (4)

Q59 fotos

- jeans (1)
- tales (2)
- sweat (3)
- photos (4)

Q60 número

- number (1)
- thing (2)
- system (3)
- course (4)

Q61 maletas

- legs (1)
- bags (2)
- fun (3)
- rows (4)

Q62 estrés

- engine (1)
- flight (2)
- stress (3)
- troops (4)

Q63 vídeos

- talks (1)
- trials (2)
- users (3)
- videos (4)

Q64 webs

- webs (1)
- pups (2)
- bono (3)
- opus (4)

Q65 fallos

- bedrooms (1)
- mistakes (2)
- counties (3)
- disputes (4)

Q66 muertes

- dreams (1)
- babies (2)
- deaths (3)
- manuals (4)

Q67 propuestas

- comments (1)
- sequences (2)
- locations (3)
- proposals (4)

Q68 citas

- appointments (1)
- descriptions (2)
- combinations (3)
- explanations (4)

Q69 agua

- seed (1)
- water (2)
- wind (3)
- base (4)

Q70 cheques

- crosses (1)
- stuffing (2)
- checks (3)
- crimes (4)

Q71 documentos

- reactions (1)
- feelings (2)
- meetings (3)
- documents (4)

Q72 dinero

- money (1)
- party (2)
- night (3)
- water (4)

Q73 cuentas

- sections (1)
- accounts (2)
- quality (3)
- schools (4)

Q74 caos

- basis (1)
- juice (2)
- chaos (3)
- wind (4)

Q75 millas

- rules (1)
- ideas (2)
- sales (3)
- miles (4)

Q76 pistola

- gun (1)
- gap (2)
- guy (3)
- bay (4)

Q77 manos

- desk (1)
- hands (2)
- bird (3)
- song (4)

Q78 cabeza

- cent (1)
- side (2)
- head (3)
- area (4)

Q79 costo

- program (1)
- authority (2)
- community (3)
- cost (4)

Q80 aviones

- airplanes (1)
- armament (2)
- behavior (3)
- adaptor (4)

Q81 besos

- grapes (1)
- kisses (2)
- trauma (3)
- jargon (4)

Q82 bebidas

- tunnel (1)
- blocks (2)
- drinks (3)
- humor (4)

Q83 partidos

- funds (1)
- calls (2)
- homes (3)
- matches (4)

Q84 equipos

- teams (1)
- fruit (2)
- steel (3)
- tears (4)

Q85 pelotas

- widows (1)
- balls (2)
- rings (3)
- angels (4)

Q86 líneas

- stands (1)
- goods (2)
- lines (3)
- trust (4)

Q87 diabetes

- hostage (1)
- balance (2)
- switch (3)
- diabetes (4)

End of Block: nouns

Start of Block: End

Q104 Thank you! This is the end of the test. 


Please let the experimenter know the test is finished.

End of Block: End
