# Supplementary material for: Individual Chunking Ability Predicts Efficient or Shallow L2 Processing: Eye-Tracking Evidence From Multiword Units in Relative Clauses
Source: Front Psychol. 2021 Jan 15;11:607621. doi: 10.3389/fpsyg.2020.607621 (PMC7844092; doi:10.3389/fpsyg.2020.607621)
Supplement: Supplementary file 4 [file Table_4.DOCX]

**Code of Linear Mixed-Effects Regression Models**

1. **Initial model for all measures**

Analyses of each region and measure (gaze or total duration) started with the initial model:

lmer(DEPENDENT VARIABLE EYE-TRACKING DATA ~

scale(Eng.Chunk.Score) + # L1 measure

scale(Sp.Chunk.Score) + # L2 measure

scale(L2.collocational.knowledge) + # L2 multiword knowledge

scale(L2.collocational.knowledge) : scale(Eng.Chunk.Score) +

scale(L2.collocational.knowledge) : scale(Sp.Chunk.Score) +

type + # type of collocation (L1-L2 congruent or incongruent)

type : scale(Eng.Chunk.Score) +

type : scale(Sp.Chunk.Score) +

type : scale(L2.collocational.knowledge) : scale(Eng.Chunk.Score) +

type : scale(L2.collocational.knowledge) : scale(Sp.Chunk.Score) +

# Covariates

scale(PSTM) + # phonological short-term memory

scale(vocab.score) + # vocabulary test

scale(log.coll.freq) + # log corpus frequency

scale(t.score) + # t-score

scale(nlength) + # noun length

scale(noun.log.freq) + # noun log frequency

scale(vlog) + # verb log frequency [for models of verb region]

scale(vlength) + # verb length [for models of verb region]

(1 + scale(trial.number) | subject) +

(1 | item),

data = DATASET,

control = lmerControl(optimizer = "bobyqa"))

1. **Selected model for noun gaze duration**

lmer(noun_gaze_duration ~

scale(Eng.Chunk.Score) + # L1 measure

scale(Sp.Chunk.Score) + # L2 measure

scale(L2.collocational.knowledge) + # L2 multiword knowledge

type + # type of collocation (L1-L2 congruent or incongruent)

(1 + scale(trial.number) | subject) +

(1 | item),

data = DATASET,

control = lmerControl(optimizer = "bobyqa"))

1. **Selected model for noun total duration**

lmer(noun_total_duration_LOG ~

scale(Eng.Chunk.Score) + # L1 measure

scale(Sp.Chunk.Score) + # L2 measure

scale(L2.collocational.knowledge) + # L2 multiword knowledge

type + # type of collocation (L1-L2 congruent or incongruent)

(1 + scale(trial.number) | subject) +

(1 | item),

data = DATASET,

control = lmerControl(optimizer = "bobyqa"))

1. **Selected model for verb gaze duration**

lmer(verb_gaze_duration ~

scale(Eng.Chunk.Score) + # L1 chunking ability

scale(Sp.Chunk.Score) + # L2 chunking ability

scale(L2.collocational.knowledge) + # L2 multiword knowledge

type + # type of collocation (L1-L2 congruent or incongruent)

scale(vocab.score) + # vocabulary test

(1 + scale(trial.number) | subject) +

(1 | item),

data = total_dur,

control = lmerControl(optimizer = "bobyqa"))

1. **Selected model for verb total duration**

lmer(total_duration_LOG ~

scale(Eng.Chunk.Score) + # L1 chunking ability

scale(Sp.Chunk.Score) + # L2 chunking ability

scale(L2.collocational.knowledge) + # L2 multiword knowledge

type + # type of collocation (L1-L2 congruent or incongruent)

scale(L2.collocational.knowledge) : scale(Sp.Chunk.Score)

type : scale(Sp.Chunk.Score) +

type : scale(L2.collocational.knowledge) : scale(Sp.Chunk.Score) +

(1 + scale(trial.number) | subject) +

(1 | item),

data = total_dur,

control = lmerControl(optimizer = "bobyqa"))

1. **Selected model for preamble total duration**

lmer(preamble_LOG ~

scale(Eng.Chunk.Score) + # L1 chunking ability

scale(Sp.Chunk.Score) + # L2 chunking ability

(1 + scale(trial.number) | subject) +

(1 | item),

data = total_dur,

control = lmerControl(optimizer = "bobyqa"))
